# Acute administration of lovastatin had no pronounced effect on motor abilities, motor coordination, gait nor simple cognition in a mouse model of Angelman syndrome

**DOI:** 10.1186/s11689-025-09616-6

**Published:** 2025-05-17

**Authors:** Timothy A. Fenton, Stela P. Petkova, Anna Adhikari, Jill L. Silverman

**Affiliations:** 1 MIND Institute, University of California Davis School of Medicine, Room 1001 A, Research II Building 96, 4625 2nd Avenue, Sacramento, CA 95817 USA; 2 Department of Psychiatry and Behavioral Sciences, University of California Davis School of Medicine, Sacramento, CA USA

**Keywords:** Lovastatin, Angelman syndrome, Behavior, Neurodevelopmental disorder, Gait, UBE3A

## Abstract

Translational research is needed to discover pharmacological targets and treatments for the diagnostic behavioral domains of neurodevelopmental disorders (NDDs), including autism spectrum disorders (ASDs) and intellectual disabilities (IDs). One NDD, associated with ASD and ID, is Angelman Syndrome (AS). AS is a rare genetic NDD for which there is currently no cure nor effective therapeutics. The genetic cause is known to be the loss of expression from the maternal allele of ubiquitin protein ligase E3A (*UBE3A*). The *Ube3a* maternal deletion mouse model of AS reliably demonstrates behavioral phenotypes of relevance to AS and therefore offers a suitable in vivo system in which to test potential therapeutics, with construct and face validity. Successes in reducing hyperexcitability and epileptogenesis have been reported in an AS model following acute treatment with lovastatin, an ERK inhibitor by reducing seizure threshold and percentage of mice exhibiting seizures. Since there has been literature reporting disruption of the ERK signaling pathway in AS, we chose to evaluate the effects of acute lovastatin administration in a tailored set of translationally relevant behavioral assays in a mouse model of AS. Unexpectedly, deleterious effects of sedation were observed in wildtype (WT), age matched littermate control mice and despite a baseline hypolocomotive phenotype in AS mice, even further reductions in exploratory activity, were observed post-acute lovastatin treatment. Limitations of this work include that chronic lower dose regimens, more akin to drug administration in humans were beyond the scope of this work, and may have produced a more favorable impact of lovastatin administration over single acute high doses. In addition, lovastatin’s effects were not assessed in younger subjects, since our study focused exclusively on adult functional outcomes. Metrics of gait, as well as motor coordination and motor learning in rotarod, previously observed to be impaired in AS mice, were not improved by lovastatin treatment. Finally, cognition by novel object recognition task was worsened in WT controls and not improved in AS, following lovastatin administration. In conclusion, lovastatin did not indicate any major improvement to AS symptoms, and in fact, worsened behavioral outcomes in the WT control groups. Therefore, despite its attractive low toxicity, immediate availability, and low cost of the drug, further investigation for clinical study is unwarranted given the results presented herein.

**Supplementary Information:**

The online version contains supplementary material available at 10.1186/s11689-025-09616-6.

## Introduction

Angelman Syndrome (AS) is a rare neurodevelopmental disorder (NDD) caused by the loss of functional ubiquitin protein ligase E3A (*UBE3A*) [1–3]. Specifically, AS results from a loss of expression from the maternal allele, leaving the brain deficient of UBE3A in most brain regions, because of neuronal-specific imprinting that silences the paternal allele with the minor exception of the superchiasmatic nucleus (SCN) [4–6] and contributions from glia, illustrated previously in AS mouse models, primates and humans [7, 8]. AS is characterized by developmental delay, intellectual disability (ID), impaired communication, gross and fine motor deficits, including movement, coordination, balance and gait, seizures and abnormal sleep [9–12]. Since these symptoms are severe and persistent, and there is currently no effective therapeutic or cure for the disorder, those with AS require lifelong supportive care. It is therefore imperative that novel strategies to treat AS are developed.

While precision therapy approaches offer future potential for disease-modifying treatments of genetically defined disorders, our understanding of these valuable therapies is that UBE3A is critical for postnatal brain development and plays a critical role in multiple neuronal signaling pathways [13]. Unfortunately, our understanding of these valuable therapies is relatively new. At the time of writing, only three gene editing therapies for neurological disorders were currently FDA approved [14]. Several molecular approaches to restore UBE3A expression in the brain are being developed including: microRNA with adeno associated viral vectors [15, 16], CRISPR/Cas9 gene therapy [17], replacement via a lentiviral replacement of a healthy *Ube3a* copy via hemopoietic stem cells [18], artificial transcription factors (ATFs) [19–22], and antisense oligonucleotides (ASOs) [23–25], to mediate paternal activation. Additional ASOs are currently being modified by industry to generate novel molecules that are being evaluated in clinical trials NCT04259281/Ultragenyx). These translational approaches have been supported by numerous basic science studies yet more research focused on the consequences of UBE3A restoration in the brain is required.

Pharmacological therapies, while not disease modifying, can have a great impact on specific symptom domains, such as motor abilities, sleep, and/or seizures, significantly improving the quality of life of individuals with AS, their families and caregivers. There is an ever-growing list of repurposed compounds that have been suggested to improve phenotypes in AS mouse models. Among these are IGF2 [26, 27], the histone deacetylation inhibitor sodium valproate [28], channel inhibitors [29], and protein phosphatase 2A inhibitor LB-100 [30]. In many rescue reports, the primary assays have been the electrophysiological rescue of long-term potentiation (LTP) in hippocampal slices. The persistent strengthening of synapses via LTP is dogmatically considered the underlying mechanism of action of learning and memory, yet many compounds that rescue LTP have not proven to be efficacious cognitive enhancers in vivo [29, 31–33].

Here we performed a tailored set of behavioral assays translationally relevant to AS, to examine doses of lovastatin in vivo. We focused on assays of motor and cognition, as these have been well reproduced clinically and are profoundly impaired clinically.

Recently, there has been wide interest in the use of statins to treat NDDs [34, 35]. Specific to AS, neurons from AS models have excessive levels of synaptic proteins, thus reducing protein synthesis with lovastatin may have clinical benefit [13, 36]. Additional rationale that guided our study of lovastatin includes its earlier success in AS mice reversing LTP impairments and improving audiogenic seizures [37]. Lovastatin also showed improvements in a variety of domains in the FXS mouse model such as reduction in both protein synthesis and audiogenic seizures [38–41] and improvements in cognition in the FXS rat model [42]. As many of these domains of behavioral deficiency in FXS overlap with AS, our laboratory assessed several doses of lovastatin as a potential therapeutic for AS in vivo using a behavioral battery relevant to AS that have been previously published [18, 22, 43–47].

Repurposing lovastatin for the treatment of AS, is an attractive prospect since it is currently widely prescribed, has been shown to be safe and well tolerated, and is approved for use in children [48]. These factors could drastically reduce the financial burden and time it takes for a drug to be used in the clinic for AS. Even if only a small subset of symptoms is alleviated, this safe, repurposed therapeutic could reduce immense hardship that individuals with AS, caregivers and their families face while waiting for a novel, curative, gene editing therapy. Thus, our objectives in this report were 1) to pharmacologically assess a variety of doses of lovastatin as a potential therapeutic for AS using an in vivo model with robust behavioral phenotypes and their WT age and sex matched controls, 2) confirm the most effective acute dose in AS models that was not deleterious *and* led to functional improvements, and 3) identify any adverse or deleterious effects of lovastatin administration in WT and sex and age matched AS experimental models.

## Materials and methods

### Mice for behavioral testing

All animals were housed in Techniplast cages (Techniplast, West Chester, PA, USA) in a temperature (68–72 °F) and humidity (~ 25%) controlled vivarium maintained on a 12:12 light–dark cycle. All procedures were approved by the Institutional Animal Care and Use Committee (IACUC) at the University of California Davis and were conducted in accordance with the National Institutes of Health Guide for the Care and Use of Laboratory Animals. B6.129S7-Ube3a^tm1 Alb/J^ mice were obtained from The Jackson Laboratory (Bar Harbor, ME, USA) and fed a standard diet of Teklad global 18% protein rodent diets 2918 (Envigo, Hayward, CA, USA). Rodent chow and tap water were available ad libitum. In addition to standard bedding, a Nestlet square, shredded brown paper, and a cardboard tube (Jonesville Corporation, Jonesville, MI) were provided in each cage. Heterozygous deletion female mice were bred with C57BL/6 J (B6 J) male mice to generate maternal deletion (*Ube3a*^−/+^, AS) and wildtype (*Ube3a*^+/+^, WT) littermates. To identify mice, pups were labeled by paw tattoo over postnatal days (PND) 2–4 using non-toxic animal tattoo ink (Ketchum Manufacturing Inc., Brockville, ON, Canada). Tails of pups were clipped (1–2 mm) for genotyping, following the UC Davis IACUC policy regarding tissue collection. Genotyping was performed with REDExtract-N-Amp (Sigma Aldrich, St. Louis, MO, USA) using primers JAX 25383 TCAATGATAGGGAGATAAAACA, 25384 GAAAACACTAACATGGAGCTC, and 25385 CTTGTGTAGCGCCAAGTGC.

To reduce carry-over effects from repeated behavioral testing, at least 48 h were allowed to pass between the completion of one task, and the start of the next task in the order of the behavioral battery [18, 26, 47, 49]. The interval between behavioral testing also allowed for the clearance of any residual lovastatin which has a half-life of 0.7–3 h [50, 51]. On days between behavioral tasks, animals were not injected. Assays were performed in the order of least to most stressful, as previously published, and between the hours of 8:00 AM PST and 7:00PM PST, during the light phase. Group sizes were chosen based on experience and power analyses [52–60].

All behavior testing was conducted by an experimenter that was blind to both genotype and treatment and included both sexes. Examining sex as a biological variable is typically, the standard practice in our laboratory. In the case of AS rodent models, our laboratory has performed analysis of sex differences over several years, across a variety of studies in AS rat and mouse models. We discovered no significant sex differences in AS rodents in the open field, the rotarod, spatial or temporal metrics of gait, the novel object recognition task, EEG, nor location discrimination and pairwise discrimination, touchscreen assays of cognition [26, 43–47, 53, 61, 62]. Other laboratories have also failed to observe sex differences in AS mice and rats, compared to WT [45, 63–66]. Cohorts were comprised of both sexes were utilized to identify any unpredicted sex effects, resulting from lovastatin treatment.

Mice were allowed to habituate in their home cages in a dimly lit room adjacent to the testing room for 1 h prior to the start of testing to limit any effect of transporting between the vivarium and testing suite. Between mice, testing apparatus surfaces were cleaned using 70% ethanol and allowed to dry. One cohort of animals was tested comprised of 14 litters beginning at 8 weeks of age (PND 55). To avoid any effect of litter, animals from each treatment group originated from between 5 and 8 different litters with no more than 3 mice from the same litter being included in any single treatment group. The order of testing was (1) open field, (2) accelerating rotarod, (3) DigiGait, (4) spontaneous alternation in the y-maze, and (5) novel object recognition. Weights of each mouse were taken prior to injection on each day that a behavior task was executed.

### Drug preparation

Lovastatin (TCI America™, L0214) was dissolved in Caprylic/Capric Triglyceride (Spectrum Chemical, C3465) at the beginning of each behavioral testing day. At the beginning of the study animals of each genotype were randomly assigned to different groups to determine which dose they would receive throughout the length of the study. Each mouse was injected IP with 10 mg/kg, 30 mg/kg, or 100 mg/kg of lovastatin or vehicle prior to performing each behavioral task. Doses were chosen based previous research in NDDs, such as Li and Silva in Neurofibromitosis-1 (NF1), Osterweil and Bear, and Muscas and Osterweil in Fragile X Syndrome (FXS), and Chung and Jiang’s report in Angelman Syndrome [37, 39, 40, 67]. To control for the half-life of lovastatin, injections were performed with staggered timing to ensure each subject was injected exactly 1 h before starting the behavioral task. In the case of multi-day tasks, subjects were injected on each day of the task, 1 h prior to beginning the task [68–71].

### Open field

General exploratory locomotion in a novel open field environment was assayed in an arena sized 40 cm × 40 cm × 30.5 cm, as previously described [22, 47, 49, 72–77]. Open field activity was considered an essential control for effects on physical activity, for example, sedation or hyperactivity which could confound the interpretation of result of interaction with objects, arena, and object exploration, and sniffing and investigation times in subsequent behavioral assays. The testing room was illuminated at ~ 40 lx.

### Rotarod

Motor coordination, balance, and motor learning were assessed using an accelerating rotarod (Ugo Basile, Schwenksville, PA) as previously described [18, 26, 47]. The task requires the mice to walk forward to remain on top of the rotating cylinder rod. Mice were given 3 trials per day with a 30–60-min inter-trial rest interval. Mice were tested over 3 consecutive days for a total of 9 trials. Latency to fall was recorded with a 300-s maximum latency.

### Gait

Treadmill gait analysis was performed using the DigiGait™ system (Mouse Specifics Inc., USA), as described [47]. Before data collection, each subject was acclimated to the Perspex walking chamber for 1 min and the treadmill was slowly accelerated to the final speed of 20 cm/s to allow mice to adjust to walking on the belt. Digital images of paw placement were recorded through a clear treadmill from the ventral plane of the animal. Mice were tested in a single session at a 20 cm/s treadmill speed maintaining a normal pace walk for *Ube3a*^+/+^ mice. Non-performers were defined as mice who were unable to sustain walking at 20 cm/s without colliding with the posterior bumper for at least 3 s. There is no practice effect, therefore, mice were allowed retrial and retest if they were unable to adjust to walking on the belt easily. The treadmill belt and the encasing Perspex chamber were cleaned with 70% (v/v) ethanol in between mice. For each mouse, videos of ∼5 s duration of all sessions were analyzed using the DigiGait™ Imaging and Analysis software v12.2 (Mouse Specifics Inc., USA). Contrast filters were determined on a mouse-by-mouse case to facilitate consistent recognition of all four paws. All analysis was conducted in a single session by an experimenter blind to genotype. Stance width (distance between paws), stride duration (time between heel strikes of the same paw), propulsion duration (time from full stance to toe push off), stance duration (time paw is in contact with the ground), stride length (distance a paw makes during a single stride) and stride frequency (number of strides per second to maintain pace) were automatically calculated. Data was averaged between left and right paws.

### Spontaneous alternation

Spontaneous alternation in a Y-maze was assayed using methods modified from previous studies [18, 26, 60, 73, 78, 79]. The Y-maze assesses spontaneous alternation and can assess short-term spatial memory [80–82]. Performance is dependent on an intact hippocampus and various interconnected structures [83]. When placed within a maze with multiple arms, mice exhibit the spontaneous behavior of alternating between arm choices with a greater frequency than re-entering the same arm most recently visited [80, 84, 85]. Our apparatus and protocol were internally validated using anticholinergics, such as scopolamine, which have shown attenuation in Y-maze alternation performance, and thus we used scopolamine as a negative control, and a task validation tool. As scopolamine is a non-specific drug, we limited inferences on “working memory.” In the absence of a proven cognitive enhancer to provide a positive control for our work, and the lack of consensus on what/when “working memory” ends, we have steered away from any controversial terminology or overinterpretation of the Y-maze assay, using “spontaneous alternation memory in the Y-maze.” Mice explored a Y- shaped maze constructed of matte white acrylic (P95 White, Tap Plastics, Sacramento, CA, USA) for 8 min and were recorded from an overhead camera with the behavioral tracking software Ethovision XT. Mice were placed at the center of the initial arm facing the center of the maze. Percentage of spontaneous alternations is calculated as the number of triads (entry into three different arms without returning to a previously entered arm) relative to the number of alternation opportunities. All scoring was conducted by an observer blind to genotype and treatment.

### Novel object recognition

The novel object recognition (NOR) test was conducted in opaque matte white (P95 White, Tap Plastics, Sacramento, CA) open field arenas (41 cm × 41 cm × 30 cm), using methods similar to those previously described [18, 49, 73–75, 86]. The experiment consisted of 4 sessions: a 30-min exposure to the open field arena the day before the test, a 10-min re-habituation on test day, a 10-min familiarization session and a 5-min recognition test. On day 1, each subject was habituated to a clean, empty, open field arena for 30-min. 24-h later, each subject was returned to the open field arena for another 10- min for the habituation phase. The mouse was then removed from the open field and placed in a clean temporary holding cage for approximately 2-min. Two identical objects were placed in the arena. Each subject was returned to the open field in which it had been habituated and allowed to freely explore for 10-min. After the familiarization session, mice were returned to their holding cages, which were transferred from the testing room to a nearby holding area for an interval of 60 min. The open field was cleaned with 70% ethanol and allowed to dry. One clean familiar object and one clean novel object were placed in the arena, where the two identical objects had been located during the familiarization phase. Each subject was returned to its open field for a 5-min recognition test, during which time it was allowed to freely explore the familiar object and the novel object. The familiarization session and the recognition test were recorded with Ethovision XT video tracking software (Version 9.0, Noldus Information Technologies, Leesburg, VA) and manually scored by an observer blinded to genotype and drug treatment. Object investigation was defined as time spent sniffing the object when the nose was oriented toward the object and the nose–object distance was 2-cm or less. Recognition memory was defined as spending significantly more time sniffing the novel object compared to the familiar object determined within genotype and within dose. Total time spent sniffing both objects was used as a measure of general exploration. Time spent sniffing two identical objects during the familiarization phase confirmed the lack of an innate side bias. Objects used were plastic toys: a small soft plastic orange safety cone and a hard plastic magnetic cone with ribbed sides, as previously described [86].

### Statistical analysis

Data were analyzed with GraphPad Prism 9 (GraphPad Software, San Diego, CA). Sex differences have not been observed in any assay, using this tailored behavioral battery for AS to date, and thus we combined sexes were utilized to achieve a powerful sample size [18, 26, 47]. Behavioral analysis passed normality distribution, using D’Agostino and Pearson tests. Data was collected using continuous variables, and thus, was analyzed via parametric analysis. Outliers were identified and excluded using Grubb’s test. Statistical testing was performed using established assay-specific methods, including two tailed student’s t-test for single parameter comparisons for spontaneous alternation and novel object recognition tasks. Where appropriate, one-way, or two-way ANOVA tests were used for open field, rotarod, and DigiGait™. As time was a variable for the open field and rotarod, two-way repeated measures ANOVA were used. Significant ANOVAs were followed by multiple comparisons, using Dunnett’s post hoc testing, when lovastatin treated groups were compared to vehicle treated groups. All significance levels were set at *p* < 0.05. Data are presented as mean ± standard error of the mean (S.E.M.) unless otherwise noted.

## Results

### Lovastatin reduced exploration in Ube3a^+/+^ (WT) and Ube3a^−/+^ (AS) mice

**Fig. 1:**
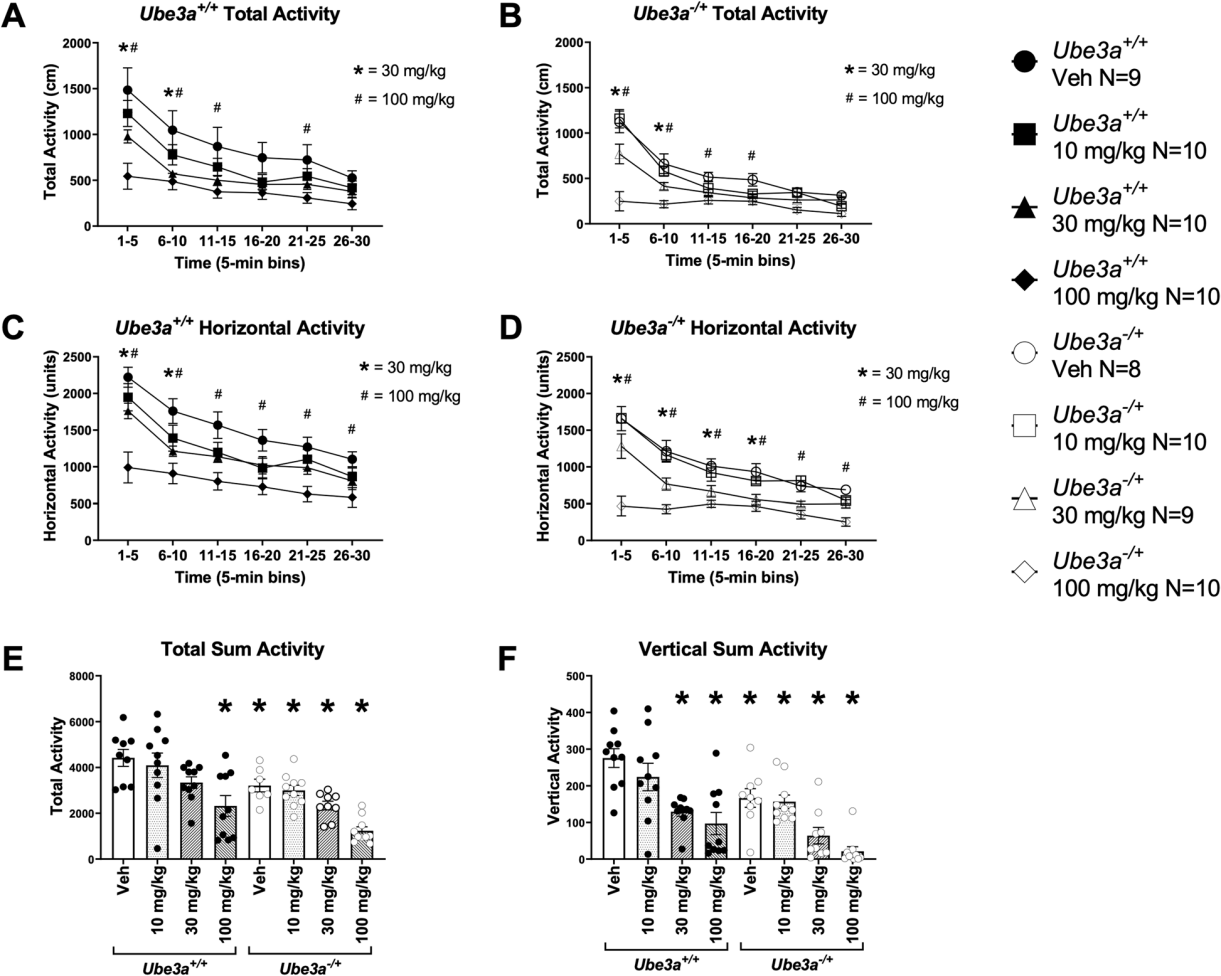
Lovastatin reduced exploration in *Ube3a*^+/+^ and *Ube3a*^−/+^ mice. **A, C** Lovastatin induced dose dependent decreases in locomotor activity in *Ube3a*^+*/*+^ mice in both (A) total activity and (C) horizontal activity, over time. Lovastatin, 30 mg/kg and 100 mg/kg reduced (**B**) total activity and (**D**) horizontal activity, over time in *Ube3a*^*−/*+^ mice. **E** Lovastatin at the 100 mg/kg dose caused a significant reduction in summed total activity across a 30-min session in both *Ube3a*^+*/*+^ and *Ube3a*^*−/*+^ mice. Lovastatin, 10 mg/kg and 30 mg/kg also reduced summed total activity in *Ube3a*^*−/*+^ mice. **F** Lovastatin, 10 mg/kg and 30 mg/kg reduced summed vertical activity (rearing) in *Ube3a*^+*/*+^ mice. *Ube3a*^*−/*+^ mice also displayed reduced summed vertical activity when treated with vehicle or any dose of lovastatin, compared to vehicle treated *Ube3a*^+*/*+^. * *p* < 0.05, A-D, two-way repeated measures ANOVA over time by 30 mg/kg of lovastatin and genotype. # *p* < 0.05, A-D, two-way repeated measures ANOVA over time by 100 mg/kg of lovastatin and genotype. * *p* < 0.05, E and F, one –way ANOVA for lovastatin, followed by Holm-Sidak post hoc analysis

Motor function is highly translational and consists of many nuanced components, including gross exploratory locomotion, movement distances, and abilities, such as balance, coordination, and gait. We utilized a tailored motor battery to perform a motor assessment that may be useful for clinical trials, which included novel locomotion in an open field arena, the accelerating rotarod, and gait analysis. As expected, we observed vehicle treated mice of both genotypes, *Ube3a*^+/+^ wildtype (WT) and *Ube3a*^−/+^ (AS), habituate to the open field arena via reduced total activity, when compared by two-way repeated measures ANOVA for genotype and lovastatin, at different doses over time (F _time x genotype x dose (35, 345)_ = 4.736, *p* < 0.0001). Lovastatin had effects in WT and AS mice (Fig. 1A; F _dose in*Ube3a*_^+/+^_(3, 36)_ = 4.156, *p* < 0.02; Fig. 1B; F _dose in*Ube3a*_^−/+^_(3, 35)_ = 4.949, *p* < 0.0005), in the total distance metric. We also observed vehicle treated mice of both genotypes habituate to the open field arena, via reduced horizontal activity, when compared by two-way repeated measures ANOVA for genotype and lovastatin, at different doses, over time (F _time x genotype x dose (35, 345)_ = 3.913, *p* < 0.0001). Genotype effects showed that AS mice differed from WT (F _genotype x dose (7, 69)_ = 13.70, *p* < 0.0001). Lovastatin had effects in WT and AS mice (Fig. 1C; F _dose in*Ube3a*_^+/+^_(3, 36)_ = 7.238, *p* < 0.0001; Fig. 1D; F _dose in*Ube3a*_^−/+^_(3, 36)_ = 11.41, *p* < 0.0005), on the horizontal activity metric. Supplementary Tables S1 and S2 illustrate the post hoc analysis. AS mice treated with vehicle traveled less distance in the open field, compared to vehicle treated WT mice (Fig. 1E; F _genotype (1, 68)_ = 20.30, *p* < 0.0001), when all time-bins were summed.

Lovastatin treatment reduced summed total activity in WT treated with 100 mg/kg of lovastatin (Fig. 1E; F _dose in*Ube3a*_^+/+^_(3, 36)_ = 1.428, *p* < 0.0001). Lovastatin also reduced summed vertical activity in WT mice (Fig. 1F; F _dose in*Ube3a*_^+/+^_(3, 36)_ = 1.981, *p* < 0.0001), treated with 30 mg/kg (*p* < 0.0005) and 100 mg/kg (*p* < 0.0001), using a one-way ANOVA, and Holm-Sidak’s post hoc analysis for multiple comparisons. Over time, and unexpectedly, detrimental effects of lovastatin were observed in WT subject mice during the total activity assessment (Fig. 1A; F _dose in*Ube3a*_^+/+^_(3, 36)_ = 4.156, *p* < 0.02). Dunnet’s post hoc analysis illustrated an adjusted *p* value of *p* = 0.001, comparing 100 mg/kg versus WT veh (Bin 1–5 min), an adjusted *p* value of *p* = 0.013, comparing 30 mg/kg versus WT veh, and *p* = 0.0027, comparing 100 mg/kg versus WT veh (Bin 6–10 min). Dunnet’s post hoc analysis illustrated an adjusted *p* value of *p* = 0.0095, comparing 100 mg/kg versus WT veh (Bin 11–15 min), and an adjusted *p* value of *p* = 0.0369, comparing 100 mg/kg versus WT veh (Bin 21–25). These data were calculated using a one-way ANOVA, and Holm-Sidak’s post hoc analysis for multiple comparisons (Supplementary Table S4).

Additionally, over time, and unexpectedly, detrimental effects of lovastatin were observed in WT subject mice when using the horizontal activity parameter (Fig. 1C; F _dose in*Ube3a*_^+/+^_(3, 36)_ = 7.238, *p* < 0.0001). Dunnet’s post hoc analysis illustrated an adjusted *p* value of *p* = 0.001, comparing 30 mg/kg versus WT veh, and *p* < 0.0001, comparing 100 mg/kg versus WT veh (Bin 1–5 min), an adjusted *p* value of *p* = 0.0065, comparing 100 mg/kg versus WT veh (Bin 6–10 min). Dunnet’s post hoc analysis illustrated an adjusted *p* value of *p* = 0.0152, comparing 100 mg/kg versus WT veh (Bin 11–15 min), an adjusted *p* value of *p* = 0.0161, comparing 100 mg/kg versus WT veh (Bin 16–20), an adjusted *p* value of *p* = 0.0082, comparing 100 mg/kg versus WT veh (Bin 21–25), and an adjusted *p* value of *p* = 0.0335, comparing 100 mg/kg versus WT veh (Bin 26–30). These data were calculated using a one-way ANOVA, and Holm-Sidak’s post hoc analysis for multiple comparisons (Supplementary Table S6).

AS mice, treated with each dose of lovastatin, (Fig. 1E; F _dose in*Ube3a*_^−/+^_(3, 36)_ = 1.185, 10 mg/kg *p* < 0.1232, 30 mg/kg *p* < 0.00097 and 100 mg/kg *p* < 0.0001), exhibited reduced summed total activity compared to vehicle treated WT mice using a one-way ANOVA, and Holm-Sidak’s post hoc analysis for multiple comparisons (Supplementary Tables S5). Genotype reduced summed vertical activity (i.e., rearing) in vehicle treated AS mice, compared to vehicle treated WT mice, (Fig. 1F; F _genotype (1, 68)_ = 19.36, *p* < 0.0001). In AS mice, every dose of lovastatin, reduced summed vertical activity (Fig. 1F; F _dose in*Ube3a*_^+/+^_(3, 34)_ = 12.96, 10 mg/kg *p* < 0.1232, 30 mg/kg *p* < 0.00097 and 100 mg/kg *p* < 0.0001), using a one-way ANOVA, and Holm-Sidak’s post hoc analysis for multiple comparisons (Supplementary Table S7).

### Lovastatin did not adversely affect rotarod performance in WT mice, but did not improve the impaired rotarod performance of AS mice

**Fig. 2:**
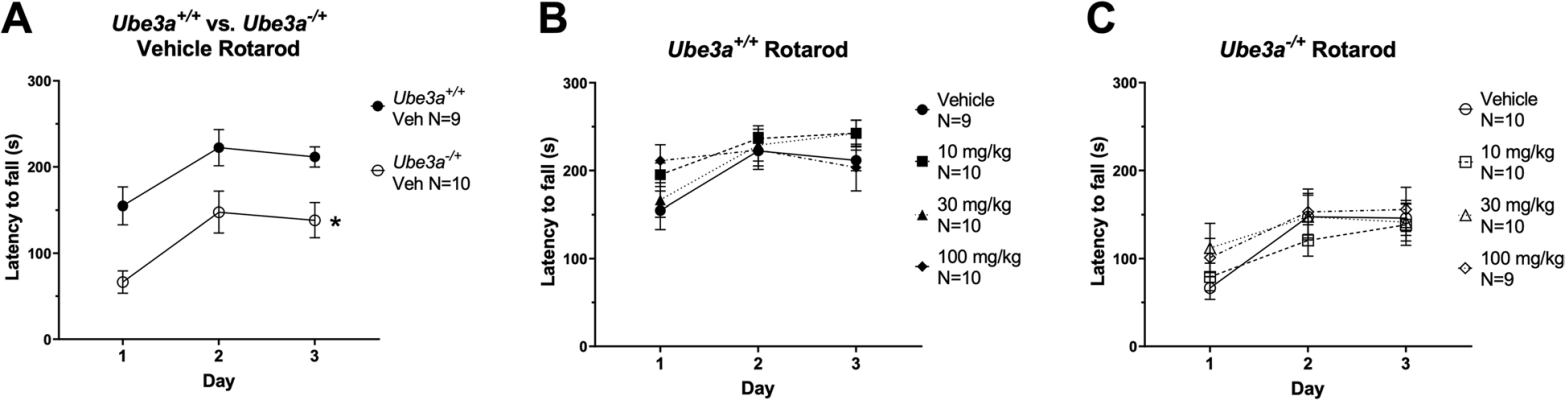
Lovastatin did not affect rotarod performance in *Ube3a*^+/+^ and *Ube3a*^−/+^ mice. **A** No significant effect of lovastatin was observed on the rotarod in *Ube3a*^+*/*+^ mice. **B** No significant effect of lovastatin was observed on the rotarod in Ube3a-/+ mice. C Vehicle treated *Ube3a*^*−/*+^ mice displayed significantly reduced latencies to fall when compared to *Ube3a*^+*/*+^ mice, at each time point, * *p* < 0.05, repeated-measures two-way ANOVA, followed by Holm-Sidak post-hoc analysis

A corroborating assay of motor abilities, coordination, and motor learning is the accelerating rotarod. We observed intact performance over time in *Ube3a*^+*/*+^ and *Ube3a*^*−/*+^ mice*,* over the three training days (Fig. 2A; F _time (2, 50)_ = 8.861, *p* < 0.0005), a genotype effect was present in the vehicle treated *Ube3a*^−/+^ group with significantly reduced latencies to fall compared to vehicle treated mice of the *Ube3a*^+/+^ group (Fig. 2A; F _genotype (1, 50)_ = 25.02, *p* < 0.0001). Training day effects were observed on the rotarod in *Ube3a*^+/+^ mice (Fig. 2B; F _time in*Ube3a*_^+/+^_(2, 105)_ = 7.967, *p* < 0.0006). No significant main effect of lovastatin was observed on the rotarod in *Ube3a*^+/+^ mice using a dose by repeated measures ANOVA statistic (Fig. 2B; F _dose x time in*Ube3a*_^+/+^_(3, 105)_ = 1.202, NS). *Ube3a*^−/+^ mutant mice displayed motor learning, via a significant main effect of training day, using a repeated measures ANOVA (Fig. 2C; F _time in*Ube3a*_^−/+^_(2, 105)_ = 7.992, *p* < 0.0006). No significant effects of lovastatin were observed on the rotarod in *Ube3a*^−/+^ mice using a dose by repeated measures ANOVA (Fig. 2C; F _dose x time in*Ube3a*_^−/+^_(3, 105)_ = 0.7801, NS).

### Lovastatin disrupted spatial and temporal gait parameters in WT mice

**Fig. 3:**
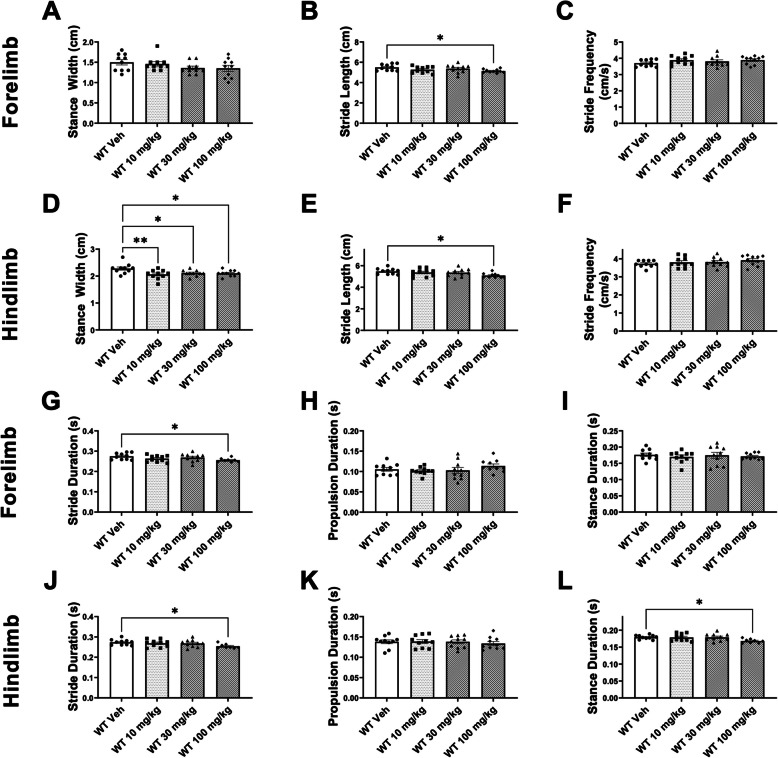
Lovastatin disrupted spatial and temporal gait parameters in *Ube3a*^+*/*+^ mice. Sedating effects of lovastatin on metrics of gait in *Ube3a*^+*/*+^ mice (WT). Lovastatin treatment at any dose did not affect (**A**) forelimb stance width, but all doses of lovastatin reduced the (**D**) hindlimb stance width. Both (**B**) forelimb stride length and (**E**) hindlimb stride length were significantly reduced when *Ube3a*^+*/*+^ mice were treated with 100 mg/kg of lovastatin. No changes were observed in (**C**) forelimb stride frequency or (**F**) hindlimb stride frequency at any dose. Reductions in (**G**) forelimb stride duration and (**J**) hindlimb stride duration were present when treated with 100 mg/kg of lovastatin. No changes were observed at any dose of lovastatin in the (**H**) forelimb propulsion duration or (**K**) hindlimb propulsion duration. Finally, a reduction in (**L**) hindlimb stance duration was observed, following 100 mg/kg of lovastatin, but no changes were observed in the (**I**) forelimb stance duration. Number of mice tested for gait: WT Veh *N* = 10, 10 mg/kg *N* = 8, 30 mg/kg *N* = 8, 100 mg/kg *N* = 7; AS Veh *N* = 9, 10 mg/kg *N* = 10, 30 mg/kg *N* = 9, 100 mg/kg *N* = 10. * *p* < 0.05, two-way ANOVA, one–way ANOVA followed by Dunnett’s post hoc analysis

We observed unexpected alterations of several spatial gait metrics, assayed using the DigiGait treadmill. Lovastatin treatment (100 mg/kg) reduced forelimb and hindlimb stride length in WT subject mice compared to vehicle treatment (Figs. 3B, E). Hindlimb stance width was reduced in WT mice, when treated with 10 mg/kg, 30 mg/kg or 100 mg/kg of lovastatin (Fig. 3D), when analyzed by a one-way ANOVA, at each dose evaluated (Fig. 3D; F _dose in*Ube3a*_^+/+^_(3, 36)_ = 2.621, *p* < 0.05), followed by Dunnett’s post hoc analysis (10 mg/kg, *p* < 0.030; 30 mg/kg, *p* < 0.040; 100 mg/kg, *p* < 0.030). In WT mice, forelimb and hindlimb stride duration were reduced by 100 mg/kg of lovastatin (Figs. 3G, J). 100 mg/kg of lovastatin, reduced forelimb (Fig. 3G; F _dose in*Ube3a*_^+/+^_(3, 36)_ = 2.377, *p* < 0.05) and hindlimb stride duration (Fig. 3J; F _dose in*Ube3a*_^+/+^_(3, 36)_ = 1.857), analyzed by a one-way ANOVA, followed by Dunnett’s post hoc analysis (100 mg/kg _forelimb_, *p* < 0.0429 and 100 mg/kg _hindlimb_, *p* < 0.0345). Hindlimb stance duration in WT mice*,* treated with 100 mg/kg of lovastatin, was also reduced (Fig. 3L; F _dose in*Ube3a*_^+/+^_(3, 36)_ = 3.614, *p* < 0.03), when compared using a one-way ANOVA, followed by Dunnett’s post hoc analysis (100 mg/kg _hindlimb stance duration_, *p* < 0.0455). Multiple parameters indicated no significant differences resulting from lovastatin treatment in forelimb and hindlimb stride frequency (Figs. 3 C, F), forelimb stance width and propulsion duration (Figs. 3A, I), and hindlimb propulsion duration (Fig. 3K).

### Lovastatin did not improve spatial and temporal gait parameters in AS mice

**Fig. 4:**
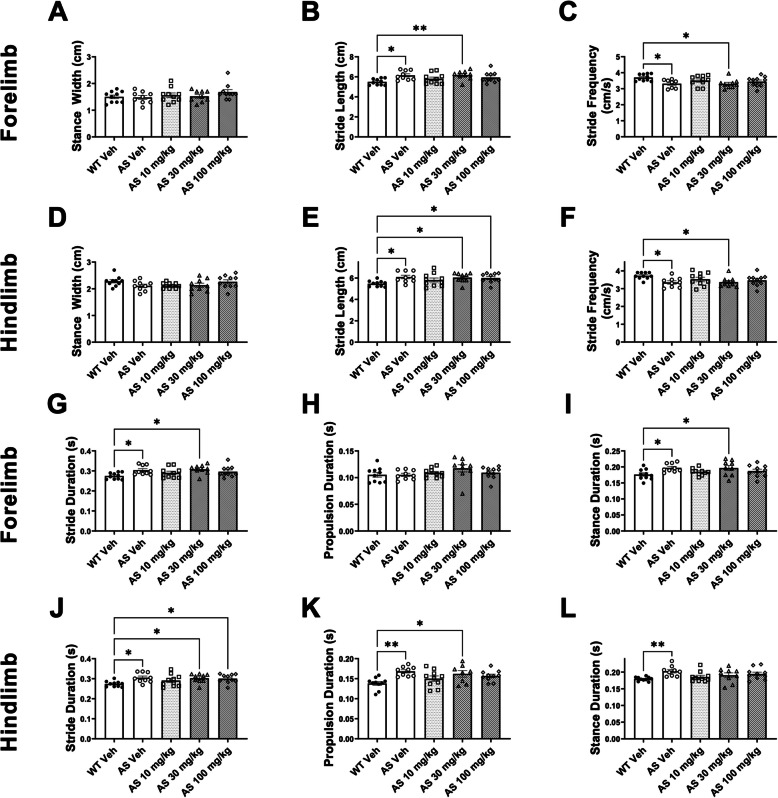
Lovastatin did not improve spatial and temporal gait parameters in *Ube3a*^*−/*+^ mice. Several metrics of gait were assessed in *Ube3a*^*−/*+^ mice (AS) treated with lovastatin. Lovastatin at any dose did not have any effect on either (**A**) forelimb stance width or (**D**) hindlimb stance width in *Ube3a*^*−/*+^ mice. However, when treated with lovastatin, reductions in forelimb stride length were observed at 10 mg/kg or 100 mg/kg (**B**) and in the (**E**) hindlimb stride length at 10 mg/kg. Similarly, a dose of either 10 mg/kg or 100 mg/kg altered both the (**C**) forelimb stride frequency and (**F**) hindlimb stride frequency, exhibiting values closer to vehicle treated *Ube3a*^+*/*+^ mice (WT). (**G**) Reductions in forelimb stride duration were observed when treated with either 10 mg/kg or 100 mg/kg of lovastatin. (**J**) Hindlimb stride duration was also reduced to levels closer to *Ube3a*^+*/*+^ mice when treated with 10 mg/kg of lovastatin. Although no changes were seen at any dose in (**H**) forelimb propulsion duration, *Ube3a*^*−/*+^ animals treated with either 10 mg/kg or 100 mg/kg displayed reductions in (**K**) hindlimb propulsion duration. Finally, (**I**) forelimb stance duration was reduced when *Ube3a*^*−/*+^ mice were treated either 10 mg/kg or 100 mg/kg of lovastatin, and (**L**) hindlimb stance duration was reduced at each dose of lovastatin evaluated. Number of mice tested for gait: WT Veh *N* = 10, 10 mg/kg *N* = 8, 30 mg/kg *N* = 8, 100 mg/kg *N* = 7; AS Veh *N* = 9, 10 mg/kg *N* = 10, 30 mg/kg *N* = 9, 100 mg/kg *N* = 10. * *p* < 0.05, two-way ANOVA, one–way ANOVA followed by Dunnett’s post hoc analysis

We observed no effects of lovastatin treatment in forelimb nor hindlimb stance width (Figs. 4A, D), nor forelimb propulsion duration (Fig. 4H) in AS mice. For forelimb and hindlimb stride length, genotype and lovastatin dose effects were identified using two-way ANOVAs (forelimb: Fig. 4B; F _genotype (1, 67)_ = 56.12, *p* < 0.001; Fig. 4B; F _dose in*Ube3a*_^−/+^_(3, 37)_ = 3.419, *p* = 0.0163 and hindlimb: Fig. 4E; F _genotype (1, 67)_ = 59.78, *p* < 0.0001; Fig. 4E; F _dose in*Ube3a*_^−/+^_(3, 37)_ = 2.912, *p* < 0.05). Vehicle treated AS mice differed from vehicle treated WT mice in forelimb and hindlimb stride length, stride frequency and stride duration (Figs. 3B, C, E, F, G, J), forelimb stance duration (Fig. 3I), and hindlimb propulsion and stance duration (Figs. 3K, L). AS mice had extended stride length, stride and stance duration, and hindlimb propulsion duration, and exhibited less stride frequency compared to WT mice. In some metrics such as stride length, lovastatin treated AS mice did not differ from vehicle treated WT mice, but also did not differ from AS vehicle mice, and thus we attributed these effects to lovastatin treatment as potentially mild, but inconclusive gait improvements. It is worth noting that differences in stride length between lovastatin treated AS mice and AS vehicle treated mice, were not detectable. Doses of 10 mg/kg (*p* = 0.5825) and 100 mg/kg (*p* = 0.2072) of lovastatin treatment in AS mice exhibited reduced forelimb stride lengths, making these AS groups indistinguishable from vehicle treated WT. Yet, despite not being significantly different from vehicle treated WT mice, differences between lovastatin treated AS mice and the forelimb stride length of the AS vehicle treated mice were not detectable, which suggests a subtle effect. 30 mg/kg, and 100 mg/kg of lovastatin treatment in AS mice resulted in extended hindlimb stride length, indistinguishable from vehicle treated AS mice, but increased compared to vehicle treated WT mice, (30 mg/kg, *p* < 0.0367 and 100 mg/kg, *p* < 0.0475). Genotype and lovastatin dose effects, respectively, were observed in forelimb and hindlimb stride frequency, using two-way ANOVAs (forelimb: Fig. 4C; F _genotype(1, 67)_ = 53.75, *p* < 0.001 and F _dose*in Ube3a*_^−/+^_(3, 67)_ = 2.855, *p* < 0.05 and hindlimb: Fig. 4F; F _genotype (1, 67)_ = 48.73, *p* < 0.001 and F _dose in*Ube3a*_^−/+^_(3, 67)_ = 2.269, *p* < 0.05). Stride frequency is reduced in AS mice compared to WT mice, by the forelimb and hindlimb indices when treated with vehicle (Fig. 4C, F). AS mice treated with 30 mg/kg of lovastatin exhibited reduced forelimb and hindlimb stride frequency compared to vehicle treated WT mice, but not versus vehicle treated AS mice following Dunnett’s post hoc analysis (30 mg/kg _forelimb_: *p* < 0.0140 and 30 mg/kg _hindlimb_: *p* < 0.0351). Although AS mice treated with 10 mg/kg or 100 mg/kg exhibit stride frequencies that are not significantly lower from vehicle treated WT mice, this fails to illustrate a robust effect of lovastatin treatment because of the lack of difference to vehicle treated AS mice. Stride length and frequency are the two metrics that have previously been reported to be altered in AS rodent models and individuals with AS by independent groups [26, 47, 87–89].

For forelimb and hindlimb, stride duration, genotype and lovastatin dose effects were identified, using two-way ANOVAs (forelimb: Fig. 4G; F _genotype(1, 67)_ = 50.81, *p* < 0.001; Fig. 4G; F _dose in*Ube3a*_^−/+^_(3, 67)_ = 2.956, *p* = 0.0304 and hindlimb: Fig. 4J; F _genotype(1, 67)_ = 54.37, *p* < 0.0001; Fig. 4J; F _dose in*Ube3a*_^−/+^_(3, 67)_ = 2.945, *p* < 0.0308). AS mice treated with 30 mg/kg of lovastatin displayed increased forelimb stride duration, analyzed with a one-way ANOVA and Dunnett’s post hoc analysis (30 mg/kg _forelimb_: *p* = 0.0124), compared to vehicle treated WT mice, but not versus the vehicle treated AS mice. AS mice treated with 30 mg/kg or 100 mg/kg of lovastatin displayed increased hindlimb stride duration, analyzed with a one-way ANOVA and Dunnett’s post hoc analysis (30 mg/kg, *p* < 0.0367 and 100 mg/kg, *p* < 0.0475), compared to vehicle treated WT mice, but not versus the vehicle treated AS mice. For forelimb and hindlimb stance duration, genotype effects were identified, using two-way ANOVAs (Fig. 4I; F _genotype (1, 67)_ = 26.50, *p* < 0.001; Fig. 4L; F _genotype (1, 67)_ = 26.88, *p* < 0.001). Lovastatin treatment at 30 mg/kg increased the forelimb stance duration in AS mice (Fig. 4I; F _dose in*Ube3a*_^−/+^_(3, 43)_ = 2.560, *p* < 0.05), by a one-way ANOVA, followed by Dunnett’s post hoc analysis (30 mg/kg _forelimb_; *p* < 0.0417), compared to vehicle treated WT mice.

Hindlimb propulsion duration exhibited a genotype effect in the vehicle groups with increased duration in AS mice compared to WT mice, with a two-way ANOVA (Fig. 4K; F _genotype (1, 67)_ = 35.10, *p* < 0.001). Lovastatin treatment at 30 mg/kg increased hindlimb propulsion duration, in AS mice (Fig. 4K; F _dose in*Ube3a*_^−/+^_(3, 43)_ = 4.355, *p* < 0.005), by a one-way ANOVA, followed by Dunnett’s post hoc analysis (30 mg/kg _forelimb_: *p* < 0.0185), compared to vehicle treated WT mice.

### Lovastatin disrupted novel object recognition in WT mice and did not improve novel object recognition in AS mice

**Fig. 5:**
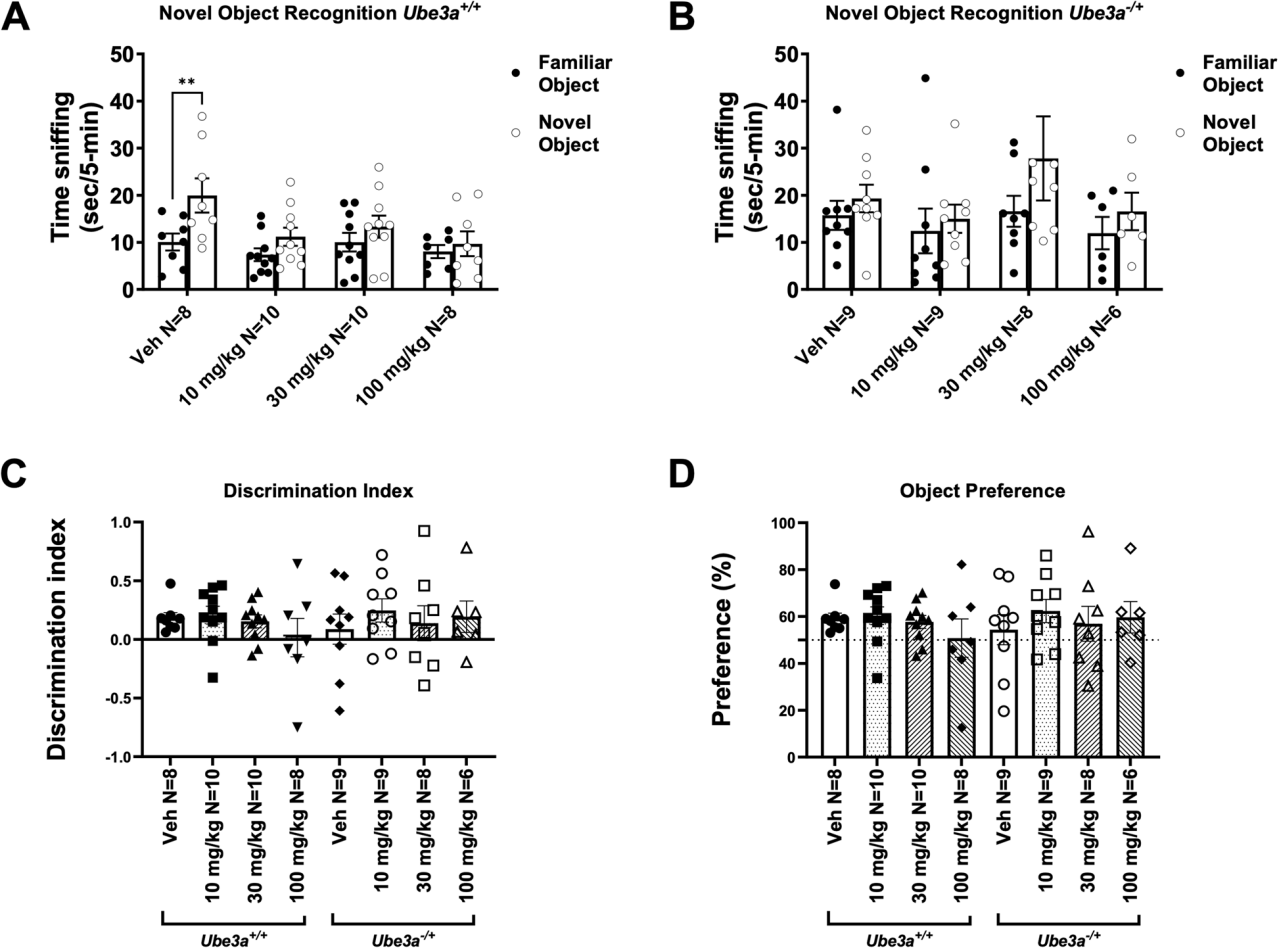
Lovastatin did not improve novel object recognition in *Ube3a*^*−/*+^ and disrupted novel object recognition in *Ube3a*^+*/*+^ mice. **A** In the novel object recognition task, vehicle treated *Ube3a*^+*/*+^ mice investigated the novel object for significantly longer than the familiar object, as expected. * *p* < 0.05, t-test of novel versus familiar. When treating *Ube3a*^+*/*+^ mice with any dose of lovastatin, the investigation time of the novel object was reduced, and no preference for investigating the novel object was observed. **B** *Ube3a*^*−/*+^ mice showed no preference for investigating the novel object when treated with vehicle or any dose of lovastatin. * *p* < 0.05, t-test of novel versus familiar object investigation. **C** Using discrimination index to measure the varying doses of lovastatin on *Ube3*^+*/*+^ and *Ube3a*^*−/*+^ mice, no significant cognitive effect was observed, via one-way ANOVA, followed by Dunnett’s posthoc comparisons. **D** Using preference ratio to measure the varying doses of lovastatin on *Ube3a*^+*/*+^ and *Ube3a*^*−/*+^ mice, no significant cognitive effect was observed, via one-way ANOVA, followed by Dunnett’s post hoc comparisons

Novel object recognition (NOR) was performed to evaluate improvement in cognition, resulting from lovastatin treatment. Vehicle treated WT mice spent more time investigating the novel object versus the familiar object, demonstrating intact recognition memory (Fig. 5A; t_(14)_ = 1.958, *p* = 0.0392), whereas vehicle treated AS mice did not (Fig. 5B; t_(16)_ = 0.8403, *p* = 0.4131), as previously published [18, 26]. Lovastatin treated WT mice disrupted these intact cognitive skills, and did not spend greater time investigating the novel object versus the familiar object, analyzed using within genotype paired t-tests (Fig. 5A; 10 mg/kg, t_(14)_ = 1.622, *p* = 0.1222; 30 mg/kg, t_(14)_ = 1.080, *p* = 0.2943; 100 mg/kg, t_(14)_ = 0.4734, *p* = 0.6438), or using discrimination index analyzed using one-way ANOVA (Fig. 5C; F _dose in*Ube3a*_^+/+^_(3, 31)_ = 0.856, *p* = 0.474), nor by preference ratio analyzed using one-way ANOVA (Fig. 5D; F _dose in*Ube3a*_^+/+^_(3, 31)_ = 0.856, *p* = 0.474), illustrating that lovastatin worsened the short term object recognition memory in WT mice, and has deleterious effects in typical control groups.

Lovastatin did not improve novel object recognition in the AS mice, illustrated by the fact that AS mice did not spend more time investigating the novel object versus the familiar object following lovastatin treatment, analyzed using within genotype paired t-tests (Fig. 5B; 10 mg/kg, t_(16)_ = 0.8403, *p* = 0.4037; 30 mg/kg, t_(16)_ = 1.181, *p* = 0.2573; 100 mg/kg, t_(14)_ = 0.8720, *p* = 0.4037), or using discrimination index analyzed using one-way ANOVA (Fig. 5C; F _dose in*Ube3a*_^−/+^_(3, 28)_ = 3.163, *p* = 0.8135), nor by preference ratio analyzed using one-way ANOVA (Fig. 5D; F _dose in*Ube3a*_^−/+^_(3, 28)_ = 3.163, *p* = 0.8135).

To rule out artifactual influence of motor activity, and behavioral changes resulting from weight differences, all mice were weighed throughout behavioral testing. Vehicle treated WT and AS mice did not exhibit significantly different weights during testing (Fig. S1 A), and no dose of lovastatin influenced weight in either subject group of WT or AS mice (Figs. S1B, S1C).

Since novel object recognition heavily relies on intact motor ability, to explore and investigate the properties of objects, the observed sedative effects in the open field, following lovastatin in the WT mice, limits the ability to make strong conclusions, regarding the cognitive aspect of novel object recognition, or the cognitive effect of lovastatin, from the adverse sedating effects of lovastatin, without additional cognitive analysis. Unfortunately, our secondary assay, spontaneous alternation in the Y-maze, is also heavily dependent on motor ability; yet more importantly, no genotype effect was observed between WT and AS mice (Figure S2). Since AS did not have a Y-maze deficit, lovastatin’s potential improvement in spontaneous alternation, as an index of cognitive performance, could not be measured via the Y-maze (Figure S2).

## Discussion

There is a large volume of preclinical and clinical knowledge around small molecule therapeutics which can be repurposed to treat the most debilitating domains of a variety of neurodevelopmental disorders (NDDs), including Angelman Syndrome (AS). For example, cannabidiol, neurosteroids, lovastatin, folic acid and betaine have been shown efficacious for seizure control in AS [37, 64, 90–92]. Despite the ease with which repurposed compounds can be evaluated in AS, non-Ube3a based therapeutic intervention has been far less studied than gene-targeted replacement-based therapies. With a vast number of compounds available and proven to be safe and well tolerated, assessing repurposed compounds, especially those with mechanisms of action up or down stream to the ubiquitination pathway, remains an unmet need.

For the first time, we report unexpectedly detrimental effects of lovastatin in WT subject mice during behavioral assays of motor abilities, including the total and horizontal activity assessments and when total activity over 30-min is summed. Furthermore, lovastatin reduced summed vertical activity in subject WT mice following 30 mg/kg and 100 mg/kg and AS subject mice treated with 10 mg/kg, 30 mg/kg and 100 mg/kg. The corroborating motor assay of motor coordination and motor learning, the accelerating rotarod, illustrated the well reported impairment in AS mice [26, 27, 44, 65, 91, 93, 94] which has been published repeatedly by us and others, while no effect of lovastatin was observed on the rotarod in either genotype.

We also discovered that numerous gait metrics that are impaired in AS, are impaired in WT mice at the 100 mg/kg dose of lovastatin. The nine sub-indices that differ between AS and WT were not fully restored by lovastatin administration across a variety of doses. The forelimb and hindlimb stride length were extended in vehicle treated AS mice and 30 mg/kg of lovastatin in AS mice caused this group to statistically differ from WT vehicle mice in forelimb stride length (Fig. 4B), while 30 mg/kg and 100 mg/kg of lovastatin in AS mice caused this group to statistically differ from WT vehicle mice in hindlimb stride length (Fig. 4E). Further, the forelimb and hindlimb stride frequency that were reduced in vehicle treated AS mice (Fig. 4C, F), were further reduced by 30 mg/kg of lovastatin in AS mice in forelimb stride frequency and 30 mg/kg and 100 mg/kg in hindlimb frequency (Fig. 4C, F). Finally, increased forelimb and hindlimb stride duration were detected in vehicle treated AS mice (Fig. 4I, L). 30 mg/kg of lovastatin in AS mice also increased forelimb stride duration and both 30 mg/kg and 100 mg/kg increased hindlimb stride duration, yet none of these alterations improved AS gait parameters to WT levels (Fig. 4G, J). AS propulsion duration did not differ in the forelimbs but the hindlimb propulsion duration was elevated, compared to WT. 30 mg/kg of lovastatin treatment in AS mice resulted in a reduction of the elevated propulsion duration, but not to a level that brought AS mouse gait to WT levels. Finally, stance duration was elevated in AS mice in both fore and hindlimb assessments, compared to WT. While 30 mg/kg of lovastatin in AS mice lowered the elevated stance duration in the forelimb index, it was not a large enough of an effect size to normalize AS stance duration to WT levels. It is worth noting a few mild gait improvements in AS mice may be attributed to lovastatin treatment but given that the effect size was so small that differences could not be observed compared to AS vehicle, any concrete interpretations are not able to be reached. Further, given the observed sedating effects of lovastatin in Fig. 1, any gait improvements on parameters that exhibited AS increases, could be confounded by the sedating effects of lovastatin.

Therefore, the preponderance of the data suggests that 100 mg/kg of lovastatin worsened gait in WT mice and that any improvements to gait in AS mice from lovastatin treatment, were either of subtle small effect sizes or may be confounded by sedation. Yet, the gait findings regarding lovastatin treatment in AS were not completely null, and our work illustrated a few subtle effects over more convincing sizeable effects.

There were no significant differences in the forelimb stance width, forelimb stride frequency, or hindlimb stride frequency in WT mice, when treated with vehicle or any dose of lovastatin (Fig. 3A, C, F). Our tailored focus on motor abilities, such as exploration, coordination and gait sharpened our observations of lovastatin, mildly rescuing some metrics with 30 mg/kg and 100 mg/kg doses of lovastatin. Rescue was demonstrated by numerous AS altered metrics, brought closer to WT levels. Stride length and frequency are two metrics that we have previously reported to be altered in AS mouse models and individuals with AS [26, 47, 87–89]. When compared to WT mice, we observed increases in stride length, stride duration, stance duration, and propulsion duration alongside reduced stride frequency in AS mice, previously observed in our lab [18, 26, 47]. Taken together, these metrics are indicative of longer, slower steps, which mimics what is seen in individuals with AS [87, 89, 95]. Stride length in the AS model averages 6–6.5 cm, with a stride frequency of approximately 3.2–3.6 steps/s, versus WT controls that exhibit stride lengths of approximately 5.2–6.0 cm and stride frequencies of 3.8–4.2 steps/s. Both 10 mg/kg and 100 mg/kg of lovastatin treatment in AS mice resulted in reduced stride lengths with an average of 5.8 cm and increased stride frequencies of approximately 4.0 steps/s, returning their values closer to WT. Y-axis values in this study reproduced earlier research using the AS mouse model [26, 47].

Gait analysis using an automated treadmill was performed using the DigiGait™ system. Quantitative gait assessments are objective, translational tools, for which automated equipment exists to acquire spatial and temporal indices in rodent models of neurodevelopmental disorders (NDDs). Similar equipment and data are being utilized, collected, and reported for clinical populations, suggesting that gait is increasing in popularity as a useful quantitative, objective outcome measure, with biomarker potential. Preclinical data have been validated by multiple laboratories, assessed across a developmental trajectory, and possess reliability and rigor by the number of metrics captured and robustness, as the findings are not specific to a piece or brand of equipment. To date, gait reports have provided preclinical data that is similar to systems for measuring human gait that utilize pressure sensitive walkways, instrumented gait analysis, and activity monitoring devices, in genetic NDDs, such as Williams, Phelan McDermid and Angelman Syndromes, as well as Neurofibromitosis-1 [47, 96–99].

Angelman Syndrome (AS) is a rare genetic disorder characterized by intellectual disabilities, motor deficits, impaired communication, and an autism spectrum disorder diagnosis. Movement disorders affect every individual with AS, regardless of the specific molecular causal subtype of the disorder. Motor impairments are the most prevalent and adverse impairment of the complex AS symptomology, which also includes recurring seizures [90, 100, 101]. Examples include spasticity, ataxia of gait (observed in most ambulatory individuals), tremors, and muscle weakness [87]. Motor behavior is highly conserved across species, exemplified by gene expression and neural circuitry, highlighting their utility as a translational outcome.

Motor deficits have been key in the study of mouse models of AS, as dysfunction on the rotarod and reduced activity have been the most consistently reported behavioral phenotypes. Clinically, motor delays and ataxia have been observed over time, and individuals with AS develop altered gait, which may signal a progressive decline in mobility. This decline is associated with decreased endurance, a more sedentary lifestyle, and diminished quality of life. Gait measurements offer a potential indicator of therapies, at a time when NDD clinical trials often lack appropriate outcomes measures. Moreover, interventions that improve gait may “spark” improvements in additional behavioral domains, offering the potential to prevent and avoid progressive declines in endurance and activity across the lifespan of those with AS [89]. This manuscript illustrates and reproduces AS mouse model deficits in overall motor ability to navigate an open field, motor coordination dysfunction on the rotarod, and gait using the automated DigiGait treadmill. However, any clear positive influence of lovastatin treatment on gait in AS mice was not evident, and the adverse influence of lovastatin treatment in WT and AS mice was observed.

Although there are no drug therapies that address the underlying causes of AS, new molecular therapies that are targeted to specific proteins, RNA, or DNA hold tremendous promise for the future. Although this strategy is promising, off-target effects and an unknown safety profile may extend the time it takes for these and other gene editing therapies to reach FDA approval and clinical use. Another promising therapeutic candidate was topotecan which reactivated the paternal allele utilizing *Ube3a*^+/YFP^ mice by inhibiting transcriptional progression of the paternal ATS [2, 65, 102, 103]. When injected unilaterally into the ventricle of mice with paternal *Ube3a-YFP*, yellow immunofluorescence from paternal expression was observed in the treated hemisphere lasting up to 12 weeks. Although topotecan was approved by the FDA for various cancers including ovarian and lung cancer [65], its lack of specificity and general toxicity [103] has limited advancement of the drug to the clinic for AS. Moreover, the intracerebroventricular route of administration is not desirable for translational pursuit, unless no other possibilities exist. Thus, while topotecan is not a viable treatment for AS, this research paved the road for future studies focusing on paternal reactivation and its mechanistic class, topoisomerase inhibitors [103], and more broadly for repurposing and small molecule therapeutics for AS.

In a preclinical study, lovastatin protected *Ube3a*^*−/*+^ mice from audiogenic seizures and reduced long burst firing in slices from these mice [37]. This suggests that there could be an anticonvulsant effect of lovastatin in AS mice. A related compound, simvastatin, also improved cognitive and social function in an AS mouse model. The authors related this effect to mechanistically restoring HDAC1/2 activity in these mice [104]. Without further evaluation, it is difficult to predict the precise effect that lovastatin would have in humans with AS, and it is possible that different routes of administration, post-treatment intervals, or dosing schemes might improve efficacy.

Lovastatin was well-tolerated in multiple clinical trials treating children with Neurofibromatosis Type 1 and some verbal and non-verbal memory improvements were observed [105, 106], however, a larger placebo-controlled trial showed no improvement in cognitive ability [107]. Clinical trials treating Fragile X Syndrome with lovastatin showed similar safety and tolerability at multiple doses and promising improvements in cognition and Aberrant Behavioral Checklist–Community (ABC-C) scores [108, 109]. Despite that, no clinical study has been conducted specifically for AS patients, the safety and efficacy results from trials of other NDDs with similar affected domains, offered promising potential for repurposing lovastatin for the treatment of AS.

In AS patients, motor skills can limit normal functions such as self-feeding, verbal ability, and cognition skills. Even if the understanding and intent is present at the communicative level, the AS patient may be unable to physically execute normal functions due to motor impairments, thus highlighting the fact that motor skills are a critical limiting factor in basic functions and any minimal improvement is highly desirable. Currently, it is our goal to identify specific parameters being used in AS clinics to identify distinct motoric and gait signatures in the *Ube3a*^−/+^ mice and rats, and identify therapeutics that improve these impairments, regardless of class or mechanism of action.

In summary, we and others [96, 97, 99] have been investigating the translatability of gait and find it to be a useful, objective, translational, cross species biomarker for preclinical models of genetic NDDs and for assessing the onset of impairments and/or progressive declines/regression in rodent models. Of additional interest in this report were the adverse effects we reported in the *Ube3a*^+/+^ mice resulting from lovastatin treatment in the open field, spatial and temporal metrics of gait and in the novel objection recognition and Y-maze learning and memory assays. Given the translatable nature of motor activity and gait analysis, the effects, that we observed when treating *Ube3a*^+/+^ and *Ube3a*^−/+^ mice with lovastatin may have clinical relevance.

## Supplementary Information

Supplementary Material 1.

## Data Availability

No datasets were generated or analysed during the current study.

## References

[1] Williams C. Angelman syndrome scientific symposium on the structure and function of UBE3A/E6AP. J Child Neurol. 2009;24(7):904–8. 10.1177/0883073809332767. (PubMed PMID: 19617463). 19617463 10.1177/0883073809332767

[2] Beaudet AL. Angelman syndrome: Drugs to awaken a paternal gene. Nature. 2011;481(7380):150–2. Epub 20111221. 10.1038/nature10784. PubMed PMID: 22190038; PMCID: PMC3638729. 22190038 10.1038/nature10784 PMC3638729

[3] Buiting K, Williams C, Horsthemke B. Angelman syndrome - insights into a rare neurogenetic disorder. Nat Rev Neurol. 2016;12(10):584–93. Epub 2016/09/13. 10.1038/nrneurol.2016.133. PubMed PMID: 27615419. 27615419 10.1038/nrneurol.2016.133

[4] Rougeulle C, Lalande M. Angelman syndrome: how many genes to remain silent? Neurogenetics. 1998;1(4):229–37. 10.1007/s100480050034. PubMed PMID: 10732796. 10732796 10.1007/s100480050034

[5] Rougeulle C, Glatt H, Lalande M. The Angelman syndrome candidate gene, UBE3A/E6-AP, is imprinted in brain. Nat Genet. 1997;17(1):14–5. 10.1038/ng0997-14. PubMed PMID: 9288088. 9288088 10.1038/ng0997-14

[6] Albrecht U, Sutcliffe JS, Cattanach BM, Beechey CV, Armstrong D, Eichele G, Beaudet AL. Imprinted expression of the murine Angelman syndrome gene, Ube3a, in hippocampal and Purkinje neurons. Nat Genet. 1997;17(1):75–8. 10.1038/ng0997-75. PubMed PMID: 9288101. 9288101 10.1038/ng0997-75

[7] Judson MC, Sosa-Pagan JO, Del Cid WA, Han JE, Philpot BD. Allelic specificity of Ube3a expression in the mouse brain during postnatal development. J Comp Neurol. 2014;522(8):1874–96. 10.1002/cne.23507.PubMedPMID:24254964;PMCID:PMC3984624. 24254964 10.1002/cne.23507 PMC3984624

[8] Burette AC, Judson MC, Li AN, Chang EF, Seeley WW, Philpot BD, Weinberg RJ. Subcellular organization of UBE3A in human cerebral cortex. Mol Autism. 2018;9:54. 10.1186/s13229-018-0238-0. Epub 20181019. PubMed PMID: 30364390; PMCID: PMC6194692. 30364390 10.1186/s13229-018-0238-0 PMC6194692

[9] Williams CA. Neurological aspects of the Angelman syndrome. Brain Dev. 2005;27(2):88–94. 10.1016/j.braindev.2003.09.014. PubMed PMID: 15668046. 15668046 10.1016/j.braindev.2003.09.014

[10] Williams CA. The behavioral phenotype of the Angelman syndrome. Am J Med Genet C Semin Med Genet. 2010;154C(4):432–7. 10.1002/ajmg.c.30278. PubMed PMID: 20981772. 20981772 10.1002/ajmg.c.30278

[11] Williams CA, Beaudet AL, Clayton-Smith J, Knoll JH, Kyllerman M, Laan LA, Magenis RE, Moncla A, Schinzel AA, Summers JA, Wagstaff J. Angelman syndrome 2005: updated consensus for diagnostic criteria. Am J Med Genet A. 2006;140(5):413–8. 10.1002/ajmg.a.31074. PubMed PMID: 16470747. 16470747 10.1002/ajmg.a.31074

[12] Williams CA, Driscoll DJ, Dagli AI. Clinical and genetic aspects of Angelman syndrome. Genet Med. 2010;12(7):385–95. 10.1097/GIM.0b013e3181def138. PubMed PMID: 20445456. 20445456 10.1097/GIM.0b013e3181def138

[13] Greer PL, Hanayama R, Bloodgood BL, Mardinly AR, Lipton DM, Flavell SW, Kim TK, Griffith EC, Waldon Z, Maehr R, Ploegh HL, Chowdhury S, Worley PF, Steen J, Greenberg ME. The Angelman Syndrome protein Ube3A regulates synapse development by ubiquitinating arc. Cell. 2010;140(5):704–16. 10.1016/j.cell.2010.01.026. PubMedPMID:20211139;PMCID:PMC2843143. 20211139 10.1016/j.cell.2010.01.026 PMC2843143

[14] Flotte TR, Gessler DJ. Gene Therapy for Rare Neurological Disorders. Clin Pharmacol Ther. 2022;111(4):743–57. 10.1002/cpt.2543. Epub 20220225. 35102556 10.1002/cpt.2543

[15] Wilson JM. Breakthrough to Bedside: Bringing Gene Therapy to Neuromuscular Diseases. Hum Gene Ther Clin Dev. 2019;30(3):93–6. 10.1089/humc.2019.29049.int. Epub 20190910. PubMed PMID: 31486676. 31486676 10.1089/humc.2019.29049.int

[16] Wilson JM. Cycling at the Frontiers of Gene Therapy. Hum Gene Ther Clin Dev. 2019;30(2):47–9. 10.1089/humc.2019.29046.int. PubMed PMID: 31215809. 31215809 10.1089/humc.2019.29046.int

[17] Wolter JM, Mao H, Fragola G, Simon JM, Krantz JL, Bazick HO, Oztemiz B, Stein JL, Zylka MJ. Cas9 gene therapy for Angelman syndrome traps Ube3a-ATS long non-coding RNA. Nature. 2020;587(7833):281–4. 10.1038/s41586-020-2835-2. Epub 20201021. PubMed PMID: 33087932; PMCID: PMC8020672. 33087932 10.1038/s41586-020-2835-2 PMC8020672

[18] Adhikari A, Copping NA, Beegle J, Cameron DL, Deng P, O’Geen H, Segal DJ, Fink KD, Silverman JL, Anderson JS. Functional rescue in an Angelman syndrome model following treatment with lentivector transduced hematopoietic stem cells. Hum Mol Genet. 2021;30(12):1067–83. 10.1093/hmg/ddab104. PubMedPMID:33856035;PMCID:PMC8188406 . 33856035 10.1093/hmg/ddab104 PMC8188406

[19] Bailus BJ, Pyles B, McAlister MM, O’Geen H, Lockwood SH, Adams AN, Nguyen JT, Yu A, Berman RF, Segal DJ. Protein Delivery of an Artificial Transcription Factor Restores Widespread Ube3a Expression in an Angelman Syndrome Mouse Brain. Mol Ther. 2016;24(3):548–55. 10.1038/mt.2015.236. Epub 20160104. PubMed PMID: 26727042; PMCID: PMC4786922. 26727042 10.1038/mt.2015.236 PMC4786922

[20] Bailus BJ, Segal DJ. The prospect of molecular therapy for Angelman syndrome and other monogenic neurologic disorders. BMC Neurosci. 2014;15:76. 10.1186/1471-2202-15-76. Epub 20140619. PubMed PMID: 24946931; PMCID: PMC4069279. 24946931 10.1186/1471-2202-15-76 PMC4069279

[21] Deng P, Halmai J, Beitnere U, Cameron D, Martinez ML, Lee CC, Waldo JJ, Thongphanh K, Adhikari A, Copping N, Petkova SP, Lee RD, Lock S, Palomares M, O’Geen H, Carter J, Gonzalez CE, Buchanan FKB, Anderson JD, Fierro FA, Nolta JA, Tarantal AF, Silverman JL, Segal DJ, Fink KD. An in vivo Cell-Based Delivery Platform for Zinc Finger Artificial Transcription Factors in Pre-clinical Animal Models. Front Mol Neurosci. 2021;14:789913. 10.3389/fnmol.2021.789913. Epub 20220127. PubMed PMID: 35153670; PMCID: PMC8829036. 35153670 10.3389/fnmol.2021.789913 PMC8829036

[22] O’Geen H, Beitnere U, Garcia MS, Adhikari A, Cameron DL, Fenton TA, Copping NA, Deng P, Lock S, Halmai J, Villegas IJ, Liu J, Wang D, Fink KD, Silverman JL, Segal DJ. Transcriptional reprogramming restores UBE3A brain-wide and rescues behavioral phenotypes in an Angelman Syndrome mouse model. Mol Ther. 2023;31(4):1088–105. 10.1016/j.ymthe.2023.01.013. Epub 20230113. PubMed PMID: 36641623. 36641623 10.1016/j.ymthe.2023.01.013 PMC10124086

[23] Meng L, Ward AJ, Chun S, Bennett CF, Beaudet AL, Rigo F. Towards a therapy for Angelman syndrome by targeting a long non-coding RNA. Nature. 2015;518(7539):409–12. 10.1038/nature13975. Epub 20141201. PubMed PMID: 25470045; PMCID: PMC4351819. 25470045 10.1038/nature13975 PMC4351819

[24] Meng L, Person RE, Huang W, Zhu PJ, Costa-Mattioli M, Beaudet AL. Truncation of Ube3a-ATS unsilences paternal Ube3a and ameliorates behavioral defects in the Angelman syndrome mouse model. PLoS Genet. 2013;9(12). 10.1371/journal.pgen.1004039. Epub 20131226. PubMed PMID: 24385930; PMCID: PMC3873245. 10.1371/journal.pgen.1004039 PMC3873245 24385930

[25] Dindot SV, Christian S, Murphy WJ, Berent A, Panagoulias J, Schlafer A, Ballard J, Radeva K, Robinson R, Myers L, Jepp T, Shaheen H, Hillman P, Konganti K, Hillhouse A, Bredemeyer KR, Black L, Douville J, Consortium F, Consortium F. An ASO therapy for Angelman syndrome that targets an evolutionarily conserved region at the start of the UBE3A-AS transcript. Sci Transl Med. 2023;15(688):4077. 10.1126/scitranslmed.abf4077. Epub 20230322. PubMed PMID: 36947593. 10.1126/scitranslmed.abf4077 36947593

[26] Berg EL, Petkova SP, Born HA, Adhikari A, Anderson AE, Silverman JL. Insulin-like growth factor-2 does not improve behavioral deficits in mouse and rat models of Angelman Syndrome. Mol Autism. 2021;12(1):59. 10.1186/s13229-021-00467-1. Epub 20210915. PubMed PMID: 34526125; PMCID: PMC8444390. 34526125 10.1186/s13229-021-00467-1 PMC8444390

[27] Cruz E, Descalzi G, Steinmetz A, Scharfman HE, Katzman A, Alberini CM. CIM6P/IGF-2 Receptor Ligands Reverse Deficits in Angelman Syndrome Model Mice. Autism Res. 2021;14(1):29–45. 10.1002/aur.2418. Epub 20201027. PubMed PMID: 33108069; PMCID: PMC8579913. 33108069 10.1002/aur.2418 PMC8579913

[28] Jamal I, Kumar V, Vatsa N, Shekhar S, Singh BK, Sharma A, Jana NR. Rescue of altered HDAC activity recovers behavioural abnormalities in a mouse model of Angelman syndrome. Neurobiol Dis. 2017;105:99–108. 10.1016/j.nbd.2017.05.010. Epub 20170530. PubMed PMID: 28576709. 28576709 10.1016/j.nbd.2017.05.010

[29] Sun J, Liu Y, Hao X, Baudry M, Bi X. Lack of UBE3A-Mediated Regulation of Synaptic SK2 Channels Contributes to Learning and Memory Impairment in the Female Mouse Model of Angelman Syndrome. Neural Plast. 2022;2022:3923384. 10.1155/2022/3923384. Epub 20221004. PubMed PMID: 36237484; PMCID: PMC9553421. 36237484 10.1155/2022/3923384 PMC9553421

[30] Wang J, Lou SS, Wang T, Wu RJ, Li G, Zhao M, Lu B, Li YY, Zhang J, Cheng X, Shen Y, Wang X, Zhu ZC, Li MJ, Takumi T, Yang H, Yu X, Liao L, Xiong ZQ. UBE3A-mediated PTPA ubiquitination and degradation regulate PP2A activity and dendritic spine morphology. Proc Natl Acad Sci U S A. 2019;116(25):12500–5. 10.1073/pnas.1820131116. Epub 20190603. PubMed PMID: 31160454; PMCID: PMC6589679. 31160454 10.1073/pnas.1820131116 PMC6589679

[31] Baudry M, Kramar E, Xu X, Zadran H, Moreno S, Lynch G, Gall C, Bi X. Ampakines promote spine actin polymerization, long-term potentiation, and learning in a mouse model of Angelman syndrome. Neurobiol Dis. 2012;47(2):210–5. 10.1016/j.nbd.2012.04.002. Epub 20120416. PubMed PMID: 22525571; PMCID: PMC3367059. 22525571 10.1016/j.nbd.2012.04.002 PMC3367059

[32] Cao C, Rioult-Pedotti MS, Migani P, Yu CJ, Tiwari R, Parang K, Spaller MR, Goebel DJ, Marshall J. Impairment of TrkB-PSD-95 signaling in Angelman syndrome. PLoS Biol. 2013;11(2):e1001478. 10.1371/journal.pbio.1001478. Epub 20130212. PubMed PMID: 23424281; PMCID: PMC3570550. 23424281 10.1371/journal.pbio.1001478 PMC3570550

[33] Liu Y, Johe K, Sun J, Hao X, Wang Y, Bi X, Baudry M. Enhancement of synaptic plasticity and reversal of impairments in motor and cognitive functions in a mouse model of Angelman Syndrome by a small neurogenic molecule, NSI-189. Neuropharmacology. 2019;144:337–44. 10.1016/j.neuropharm.2018.10.038. Epub 20181105. PubMed PMID: 30408487. 30408487 10.1016/j.neuropharm.2018.10.038

[34] Mouro FM, Miranda-Lourenco C, Sebastiao AM, Diogenes MJ. From Cannabinoids and Neurosteroids to Statins and the Ketogenic Diet: New Therapeutic Avenues in Rett Syndrome? Front Neurosci. 2019;13:680. 10.3389/fnins.2019.00680. Epub 20190702. PubMed PMID: 31333401; PMCID: PMC6614559. 31333401 10.3389/fnins.2019.00680 PMC6614559

[35] Ottenhoff MJ, Krab LC, Elgersma Y. Considerations for Clinical Therapeutic Development of Statins for Neurodevelopmental Disorders. eNeuro. 2020;7(2)ENEURO.0392-19.2020. 10.1523/ENEURO.0392-19.2020. Epub 20200306. PubMed PMID: 32071072; PMCID: PMC7070444. 10.1523/ENEURO.0392-19.2020 PMC7070444 32071072

[36] Aria F, Pandey K, Alberini CM. Excessive Protein Accumulation and Impaired Autophagy in the Hippocampus of Angelman Syndrome Modeled in Mice. Biol Psychiatry. 2023;94(1):68–83. 10.1016/j.biopsych.2022.11.016. Epub 20221205. PubMed PMID: 36764852; PMCID: PMC10276539. 36764852 10.1016/j.biopsych.2022.11.016 PMC10276539

[37] Chung L, Bey AL, Towers AJ, Cao X, Kim IH, Jiang YH. Lovastatin suppresses hyperexcitability and seizure in Angelman syndrome model. Neurobiol Dis. 2018;110:12–9. 10.1016/j.nbd.2017.10.016. Epub 20171031. PubMed PMID: 29097328; PMCID: PMC5903876. 29097328 10.1016/j.nbd.2017.10.016 PMC5903876

[38] Muscas M, Seo SS, Louros SR, Osterweil EK. A Differential Effect of Lovastatin versus Simvastatin in Neurodevelopmental Disorders. eNeuro. 2020;7(4):ENEURO.0162-20.2020. 10.1523/ENEURO.0162-20.2020. Epub 20200813. PubMed PMID: 32651266; PMCID: PMC7433894. 10.1523/ENEURO.0162-20.2020 PMC7433894 32651266

[39] Muscas M, Louros SR, Osterweil EK. Lovastatin, not Simvastatin, Corrects Core Phenotypes in the Fragile X Mouse Model. eNeuro. 2019;6(3):ENEURO.0097-19.2019. 10.1523/ENEURO.0097-19.2019. Epub 20190612. PubMed PMID: 31147392; PMCID: PMC6565377. 10.1523/ENEURO.0097-19.2019 PMC6565377 31147392

[40] Osterweil EK, Chuang SC, Chubykin AA, Sidorov M, Bianchi R, Wong RK, Bear MF. Lovastatin corrects excess protein synthesis and prevents epileptogenesis in a mouse model of fragile X syndrome. Neuron. 2013;77(2):243–50. 10.1016/j.neuron.2012.01.034. PubMedPMID:23352161;PMCID:PMC3597444. 23352161 10.1016/j.neuron.2012.01.034 PMC3597444

[41] Pellerin D, Caku A, Fradet M, Bouvier P, Dube J, Corbin F. Lovastatin corrects ERK pathway hyperactivation in fragile X syndrome: potential of platelet’s signaling cascades as new outcome measures in clinical trials. Biomarkers. 2016;21(6):497–508. 10.3109/1354750X.2016.1160289. Epub 20160408. PubMed PMID: 27058300. 27058300 10.3109/1354750X.2016.1160289

[42] Asiminas A, Jackson AD, Louros SR, Till SM, Spano T, Dando O, Bear MF, Chattarji S, Hardingham GE, Osterweil EK, Wyllie DJA, Wood ER, Kind PC. Sustained correction of associative learning deficits after brief, early treatment in a rat model of Fragile X Syndrome. Sci Transl Med. 2019;11(494):eaao0498. 10.1126/scitranslmed.aao0498. PubMed PMID: 31142675; PMCID: PMC8162683. 31142675 10.1126/scitranslmed.aao0498 PMC8162683

[43] Berg EL, Jami SA, Petkova SP, Berz A, Fenton TA, Lerch JP, Segal DJ, Gray JA, Ellegood J, Wohr M, Silverman JL. Excessive Laughter-like Vocalizations, Microcephaly, and Translational Outcomes in the Ube3a Deletion Rat Model of Angelman Syndrome. J Neurosci. 2021;41(42):8801–14. 10.1523/JNEUROSCI.0925-21.2021. Epub 20210902. PubMed PMID: 34475199; PMCID: PMC8528495. 34475199 10.1523/JNEUROSCI.0925-21.2021 PMC8528495

[44] Berg EL, Pride MC, Petkova SP, Lee RD, Copping NA, Shen Y, Adhikari A, Fenton TA, Pedersen LR, Noakes LS, Nieman BJ, Lerch JP, Harris S, Born HA, Peters MM, Deng P, Cameron DL, Fink KD, Beitnere U, O'Geen H, Anderson AE, Dindot SV, Nash KR, Weeber EJ, Wohr M, Ellegood J, Segal DJ, Silverman JL. Translational outcomes in a full gene deletion of ubiquitin protein ligase E3A rat model of Angelman syndrome. Transl Psychiatry. 2020;10(1):39. Epub 2020/02/19. 10.1038/s41398-020-0720-2. PubMed PMID: 32066685; PMCID: PMC7026078. 10.1038/s41398-020-0720-2 PMC7026078 32066685

[45] Born HA, Martinez LA, Levine AT, Harris SE, Mehra S, Lee WL, Dindot SV, Nash KR, Silverman JL, Segal DJ, Weeber EJ, Anderson AE. Early Developmental EEG and Seizure Phenotypes in a Full Gene Deletion of Ubiquitin Protein Ligase E3A Rat Model of Angelman Syndrome. eNeuro. 2021;8(2). Epub 20210324. 10.1523/ENEURO.0345-20.2020. PubMed PMID: 33531368; PMCID: PMC8114899. 10.1523/ENEURO.0345-20.2020 PMC8114899 33531368

[46] Copping NA, Silverman JL. Abnormal electrophysiological phenotypes and sleep deficits in a mouse model of Angelman Syndrome. Mol Autism. 2021;12(1):9. Epub 2021/02/08. 10.1186/s13229-021-00416-y. PubMed PMID: 33549123; PMCID: PMC7866697. 10.1186/s13229-021-00416-y PMC7866697 33549123

[47] Petkova SP, Adhikari A, Berg EL, Fenton TA, Duis J, Silverman JL. Gait as a quantitative translational outcome measure in Angelman syndrome. Autism Res. 2022;15(5):821–33. Epub 20220310. 10.1002/aur.2697. PubMed PMID: 35274462; PMCID: PMC9311146. 10.1002/aur.2697 PMC9311146 35274462

[48] Mainberger F, Langer S, Mall V, Jung NH. Impaired synaptic plasticity in RASopathies: a mini-review. J Neural Transm (Vienna). 2016;123(10):1133–8. Epub 20160826. 10.1007/s00702-016-1609-3. PubMed PMID: 27565148. 10.1007/s00702-016-1609-3 27565148

[49] Copping NA, Christian SGB, Ritter DJ, Islam MS, Buscher N, Zolkowska D, Pride MC, Berg EL, LaSalle JM, Ellegood J, Lerch JP, Reiter LT, Silverman JL, Dindot SV. Neuronal overexpression of Ube3a isoform 2 causes behavioral impairments and neuroanatomical pathology relevant to 15q11.2-q13.3 duplication syndrome. Hum Mol Genet. 2017;26(20):3995–4010. 10.1093/hmg/ddx289. PubMed PMID: 29016856; PMCID: PMC5886211. 10.1093/hmg/ddx289 PMC5886211 29016856

[50] Tomaszewski M, Zolkowska D, Plewa Z, Czuczwar SJ, Luszczki JJ. Effect of acute and chronic exposure to lovastatin on the anticonvulsant action of classical antiepileptic drugs in the mouse maximal electroshock-induced seizure model. Eur J Pharmacol. 2021;907:174290. Epub 20210701. 10.1016/j.ejphar.2021.174290. PubMed PMID: 34217711. 10.1016/j.ejphar.2021.174290 34217711

[51] Zhu Y, D'Agostino J, Zhang QY. Role of intestinal cytochrome P450 (P450) in modulating the bioavailability of oral lovastatin: insights from studies on the intestinal epithelium-specific P450 reductase knockout mouse. Drug Metab Dispos. 2011;39(6):939–43. Epub 20110224. 10.1124/dmd.110.037861. PubMed PMID: 21349922; PMCID: PMC3100899. 10.1124/dmd.110.037861 PMC3100899 21349922

[52] Silverman JL, Crawley JN. The promising trajectory of autism therapeutics discovery. Drug Discov Today. 2014;19(7):838–44. Epub 20131218. 10.1016/j.drudis.2013.12.007. PubMed PMID: 24362109. 10.1016/j.drudis.2013.12.007 24362109

[53] Kazdoba TM, Leach PT, Yang M, Silverman JL, Solomon M, Crawley JN. Translational Mouse Models of Autism: Advancing Toward Pharmacological Therapeutics. Curr Top Behav Neurosci. 2016;28:1–52. 10.1007/7854_2015_5003.PubMedPMID:27305922;PMCID:PMC5116923. 27305922 10.1007/7854_2015_5003 PMC5116923

[54] Silverman JL, Nithianantharajah J, Der-Avakian A, Young JW, Sukoff Rizzo SJ. Lost in translation: At the crossroads of face validity and translational utility of behavioral assays in animal models for the development of therapeutics. Neurosci Biobehav Rev. 2020;116:452–3. Epub 2020/07/19. 10.1016/j.neubiorev.2020.07.008. PubMed PMID: 32681939. 10.1016/j.neubiorev.2020.07.008 PMC7773218 32681939

[55] Sukoff Rizzo SJ, Silverman JL. Methodological Considerations for Optimizing and Validating Behavioral Assays. Curr Protoc Mouse Biol. 2016;6(4):364–79. Epub 20161201. 10.1002/cpmo.17. PubMed PMID: 27906464; PMCID: PMC6054129. 10.1002/cpmo.17 PMC6054129 27906464

[56] Sukoff Rizzo SJ, Homanics G, Schaeffer DJ, Schaeffer L, Park JE, Oluoch J, Zhang T, Haber A, Seyfried NT, Paten B, Greenwood A, Murai T, Choi SH, Huhe H, Kofler J, Strick PL, Carter GW, Silva AC. Bridging the rodent to human translational gap: Marmosets as model systems for the study of Alzheimer's disease. Alzheimers Dement (N Y). 2023;9(3):e12417. Epub 20230821. 10.1002/trc2.12417. PubMed PMID: 37614242; PMCID: PMC10442521. 10.1002/trc2.12417 PMC10442521 37614242

[57] Sukoff Rizzo SJ. The essential role of animal models in the advancement of our understanding of human behaviors: A Commentary on the Special issue on the 30th Anniversary of the International Behavioral Neuroscience Society (IBNS). Neurosci Biobehav Rev. 2023;149:105182. Epub 20230417. 10.1016/j.neubiorev.2023.105182. PubMed PMID: 37076055. 10.1016/j.neubiorev.2023.105182 37076055

[58] Sukoff Rizzo SJ, Masters A, Onos KD, Quinney S, Sasner M, Oblak A, Lamb BT, Territo PR, consortium M-A. Improving preclinical to clinical translation in Alzheimer's disease research. Alzheimers Dement (N Y). 2020;6(1):e12038. Epub 20200614. 10.1002/trc2.12038. PubMed PMID: 32548237; PMCID: PMC7293992. 10.1002/trc2.12038 PMC7293992 32548237

[59] Sukoff Rizzo SJ, McTighe S, McKinzie DL. Genetic Background and Sex: Impact on Generalizability of Research Findings in Pharmacology Studies. Handb Exp Pharmacol. 2020;257:147–62. 10.1007/164_2019_282. PubMed PMID: 31595415. 31595415 10.1007/164_2019_282

[60] Sukoff Rizzo SJ, Anderson LC, Green TL, McGarr T, Wells G, Winter SS. Assessing Healthspan and Lifespan Measures in Aging Mice: Optimization of Testing Protocols, Replicability, and Rater Reliability. Curr Protoc Mouse Biol. 2018;8(2):e45. Epub 2018/06/21. 10.1002/cpmo.45. PubMed PMID: 29924918. 10.1002/cpmo.45 29924918

[61] Dodge A, Peters MM, Greene HE, Dietrick C, Botelho R, Chung D, Willman J, Nenninger AW, Ciarlone S, Kamath SG, Houdek P, Sumova A, Anderson AE, Dindot SV, Berg EL, O'Geen H, Segal DJ, Silverman JL, Weeber EJ, Nash KR. Generation of a Novel Rat Model of Angelman Syndrome with a Complete Ube3a Gene Deletion. Autism Res. 2020;13(3):397–409. Epub 20200121. 10.1002/aur.2267. PubMed PMID: 31961493; PMCID: PMC7787396. 10.1002/aur.2267 PMC7787396 31961493

[62] Leach PT, Hayes J, Pride M, Silverman JL, Crawley JN. Normal Performance of Fmr1 Mice on a Touchscreen Delayed Nonmatching to Position Working Memory Task. eNeuro. 2016;3(1). Epub 20160315. 10.1523/ENEURO.0143-15.2016. PubMed PMID: 27022628; PMCID: PMC4800045. 10.1523/ENEURO.0143-15.2016 PMC4800045 27022628

[63] Sidorov MS, Judson MC, Kim H, Rougie M, Ferrer AI, Nikolova VD, Riddick NV, Moy SS, Philpot BD. Enhanced Operant Extinction and Prefrontal Excitability in a Mouse Model of Angelman Syndrome. J Neurosci. 2018;38(11):2671–82. Epub 20180205. 10.1523/JNEUROSCI.2828-17.2018. PubMed PMID: 29431654; PMCID: PMC5852653. 10.1523/JNEUROSCI.2828-17.2018 PMC5852653 29431654

[64] Gu B, Zhu M, Glass MR, Rougie M, Nikolova VD, Moy SS, Carney PR, Philpot BD. Cannabidiol attenuates seizures and EEG abnormalities in Angelman syndrome model mice. J Clin Invest. 2019;129(12):5462–7. 10.1172/JCI130419.PubMedPMID:31503547;PMCID:PMC6877312. 31503547 10.1172/JCI130419 PMC6877312

[65] Huang HS, Burns AJ, Nonneman RJ, Baker LK, Riddick NV, Nikolova VD, Riday TT, Yashiro K, Philpot BD, Moy SS. Behavioral deficits in an Angelman syndrome model: effects of genetic background and age. Behav Brain Res. 2013;243:79–90. Epub 20130104. 10.1016/j.bbr.2012.12.052. PubMed PMID: 23295389; PMCID: PMC3629944. 10.1016/j.bbr.2012.12.052 PMC3629944 23295389

[66] Martinez LA, Born HA, Harris S, Regnier-Golanov A, Grieco JC, Weeber EJ, Anderson AE. Quantitative EEG Analysis in Angelman Syndrome: Candidate Method for Assessing Therapeutics. Clin EEG Neurosci. 2020:1550059420973095. Epub 20201118. 10.1177/1550059420973095. PubMed PMID: 33203220. 10.1177/1550059420973095 33203220

[67] Li W, Cui Y, Kushner SA, Brown RA, Jentsch JD, Frankland PW, Cannon TD, Silva AJ. The HMG-CoA reductase inhibitor lovastatin reverses the learning and attention deficits in a mouse model of neurofibromatosis type 1. Curr Biol. 2005;15(21):1961–7. 10.1016/j.cub.2005.09.043. PubMed PMID: 16271875. 16271875 10.1016/j.cub.2005.09.043

[68] Chen Y, Li LB, Zhang J, Tang DP, Wei JJ, Zhuang ZH. Simvastatin, but not pravastatin, inhibits the proliferation of esophageal adenocarcinoma and squamous cell carcinoma cells: a cell-molecular study. Lipids Health Dis. 2018;17(1):290. Epub 20181222. 10.1186/s12944-018-0946-7. PubMed PMID: 30579354; PMCID: PMC6303879. 10.1186/s12944-018-0946-7 PMC6303879 30579354

[69] Ongerth T, Russmann V, Fischborn S, Boes K, Siegl C, Potschka H. Targeting of microglial KCa3.1 channels by TRAM-34 exacerbates hippocampal neurodegeneration and does not affect ictogenesis and epileptogenesis in chronic temporal lobe epilepsy models. Eur J Pharmacol. 2014;740:72–80. Epub 20140710. 10.1016/j.ejphar.2014.06.061. PubMed PMID: 25016931. 10.1016/j.ejphar.2014.06.061 25016931

[70] Glaser N, Chu S, Weiner J, Zdepski L, Wulff H, Tancredi D, ME OD. Effects of TRAM-34 and minocycline on neuroinflammation caused by diabetic ketoacidosis in a rat model. BMJ Open Diabetes Res Care. 2022;10(3). 10.1136/bmjdrc-2022-002777. PubMed PMID: 35584854; PMCID: PMC9119135. 10.1136/bmjdrc-2022-002777 PMC9119135 35584854

[71] Glaser N, Little C, Lo W, Cohen M, Tancredi D, Wulff H, O'Donnell M. Treatment with the KCa3.1 inhibitor TRAM-34 during diabetic ketoacidosis reduces inflammatory changes in the brain. Pediatr Diabetes. 2017;18(5):356–66. Epub 20160513. 10.1111/pedi.12396. PubMed PMID: 27174668. 10.1111/pedi.12396 27174668

[72] Dhamne SC, Silverman JL, Super CE, Lammers SHT, Hameed MQ, Modi ME, Copping NA, Pride MC, Smith DG, Rotenberg A, Crawley JN, Sahin M. Replicable in vivo physiological and behavioral phenotypes of the Shank3B null mutant mouse model of autism. Mol Autism. 2017;8:26. 10.1186/s13229-017-0142-z.PubMedPMID:28638591;PMCID:PMC5472997. 28638591 10.1186/s13229-017-0142-z PMC5472997

[73] Gompers AL, Su-Feher L, Ellegood J, Copping NA, Riyadh MA, Stradleigh TW, Pride MC, Schaffler MD, Wade AA, Catta-Preta R, Zdilar I, Louis S, Kaushik G, Mannion BJ, Plajzer-Frick I, Afzal V, Visel A, Pennacchio LA, Dickel DE, Lerch JP, Crawley JN, Zarbalis KS, Silverman JL, Nord AS. Germline Chd8 haploinsufficiency alters brain development in mouse. Nat Neurosci. 2017;20(8):1062–73. Epub 20170626. 10.1038/nn.4592. PubMed PMID: 28671691; PMCID: PMC6008102. 10.1038/nn.4592 PMC6008102 28671691

[74] Haigh JL, Adhikari A, Copping NA, Stradleigh T, Wade AA, Catta-Preta R, Su-Feher L, Zdilar I, Morse S, Fenton TA, Nguyen A, Quintero D, Agezew S, Sramek M, Kreun EJ, Carter J, Gompers A, Lambert JT, Canales CP, Pennacchio LA, Visel A, Dickel DE, Silverman JL, Nord AS. Deletion of a non-canonical regulatory sequence causes loss of Scn1a expression and epileptic phenotypes in mice. Genome Med. 2021;13(1):69. Epub 20210426. 10.1186/s13073-021-00884-0. PubMed PMID: 33910599; PMCID: PMC8080386. 10.1186/s13073-021-00884-0 PMC8080386 33910599

[75] Silverman JL, Fenton T, Haouchine O, Hallam E, Smith E, Jackson K, Rahbarian D, Canales C, Adhikari A, Nord A, Ben-Shalom R. Hyperexcitability and translational phenotypes in a preclinical model of SYNGAP1 mutations. Res Sq. 2023. Epub 20230913. 10.21203/rs.3.rs-3246655/v1. PubMed PMID: 37790402; PMCID: PMC10543290.

[76] Silverman JL, Pride MC, Hayes JE, Puhger KR, Butler-Struben HM, Baker S, Crawley JN. GABAB Receptor Agonist R-Baclofen Reverses Social Deficits and Reduces Repetitive Behavior in Two Mouse Models of Autism. Neuropsychopharmacology. 2015;40(9):2228–39. Epub 20150310. 10.1038/npp.2015.66. PubMed PMID: 25754761; PMCID: PMC4613612. 10.1038/npp.2015.66 PMC4613612 25754761

[77] Silverman JL, Smith DG, Rizzo SJ, Karras MN, Turner SM, Tolu SS, Bryce DK, Smith DL, Fonseca K, Ring RH, Crawley JN. Negative allosteric modulation of the mGluR5 receptor reduces repetitive behaviors and rescues social deficits in mouse models of autism. Sci Transl Med. 2012;4(131):131ra51. 10.1126/scitranslmed.3003501. PubMed PMID: 22539775; PMCID: PMC4904784. 10.1126/scitranslmed.3003501 PMC4904784 22539775

[78] Adhikari A, Buchanan FKB, Fenton TA, Cameron DL, Halmai J, Copping NA, Fink KD, Silverman JL. Touchscreen cognitive deficits, hyperexcitability and hyperactivity in males and females using two models of Cdkl5 deficiency. Hum Mol Genet. 2022;31(18):3032–50. 10.1093/hmg/ddac091.PubMedPMID:35445702;PMCID:PMC9476626. 35445702 10.1093/hmg/ddac091 PMC9476626

[79] Fenton TA, Haouchine OY, Hallam EL, Smith EM, Jackson KC, Rahbarian D, Canales C, Adhikari A, Nord AS, Ben-Shalom R, Silverman JL. Hyperexcitability and translational phenotypes in a preclinical model of SYNGAP1 mutations. bioRxiv. 2023. Epub 20230726. 10.1101/2023.07.24.550093. PubMed PMID: 37546838; PMCID: PMC10402099.

[80] Hughes RN. The value of spontaneous alternation behavior (SAB) as a test of retention in pharmacological investigations of memory. Neurosci Biobehav Rev. 2004;28(5):497–505. 10.1016/j.neubiorev.2004.06.006. PubMed PMID: 15465137. 15465137 10.1016/j.neubiorev.2004.06.006

[81] Kokkinidis L, Anisman H. Dissociation of the effects of scopolamine and d-amphetamine on a spontaneous alternation task. Pharmacol Biochem Behav. 1976;5(3):293–7. 10.1016/0091-3057(76)90081-2. PubMed PMID: 996062. 996062 10.1016/0091-3057(76)90081-2

[82] Prieur EAK, Jadavji NM. Assessing Spatial Working Memory Using the Spontaneous Alternation Y-maze Test in Aged Male Mice. Bio Protoc. 2019;9(3):e3162. Epub 20190205. 10.21769/BioProtoc.3162. PubMed PMID: 33654968; PMCID: PMC7854095. 10.21769/BioProtoc.3162 PMC7854095 33654968

[83] Lalonde R, Dumont M, Fukuchi K, Strazielle C. Transgenic mice expressing the human C99 terminal fragment of betaAPP: effects on spatial learning, exploration, anxiety, and motor coordination. Exp Gerontol. 2002;37(12):1401–12. 10.1016/s0531-5565(02)00123-7. PubMed PMID: 12559409. 12559409 10.1016/s0531-5565(02)00123-7

[84] Lainiola M, Procaccini C, Linden AM. mGluR3 knockout mice show a working memory defect and an enhanced response to MK-801 in the T- and Y-maze cognitive tests. Behav Brain Res. 2014;266:94–103. Epub 20140311. 10.1016/j.bbr.2014.03.008. PubMed PMID: 24631392. 10.1016/j.bbr.2014.03.008 24631392

[85] Kim J, Kang H, Lee YB, Lee B, Lee D. A quantitative analysis of spontaneous alternation behaviors on a Y-maze reveals adverse effects of acute social isolation on spatial working memory. Sci Rep. 2023;13(1):14722. Epub 20230907. 10.1038/s41598-023-41996-4. PubMed PMID: 37679447; PMCID: PMC10485067. 10.1038/s41598-023-41996-4 PMC10485067 37679447

[86] Gulinello M, Mitchell HA, Chang Q, Timothy O'Brien W, Zhou Z, Abel T, Wang L, Corbin JG, Veeraragavan S, Samaco RC, Andrews NA, Fagiolini M, Cole TB, Burbacher TM, Crawley JN. Rigor and reproducibility in rodent behavioral research. Neurobiol Learn Mem. 2019;165:106780. Epub 20180104. 10.1016/j.nlm.2018.01.001. PubMed PMID: 29307548; PMCID: PMC6034984. 10.1016/j.nlm.2018.01.001 PMC6034984 29307548

[87] Grieco JC, Gouelle A, Weeber EJ. Identification of spatiotemporal gait parameters and pressure-related characteristics in children with Angelman syndrome: A pilot study. J Appl Res Intellect Disabil. 2018;31(6):1219–24. Epub 20180508. 10.1111/jar.12462. PubMed PMID: 29737626. 10.1111/jar.12462 29737626

[88] Duis J, Nespeca M, Summers J, Bird L, Bindels-de Heus K, Valstar MJ, de Wit MY, Navis C, Ten Hooven-Radstaake M, van Iperen-Kolk BM, Ernst S, Dendrinos M, Katz T, Diaz-Medina G, Katyayan A, Nangia S, Thibert R, Glaze D, Keary C, Pelc K, Simon N, Sadhwani A, Heussler H, Wheeler A, Woeber C, DeRamus M, Thomas A, Kertcher E, DeValk L, Kalemeris K, Arps K, Baym C, Harris N, Gorham JP, Bohnsack BL, Chambers RC, Harris S, Chambers HG, Okoniewski K, Jalazo ER, Berent A, Bacino CA, Williams C, Anderson A. A multidisciplinary approach and consensus statement to establish standards of care for Angelman syndrome. Mol Genet Genomic Med. 2022;10(3):e1843. Epub 20220211. 10.1002/mgg3.1843. PubMed PMID: 35150089; PMCID: PMC8922964. 10.1002/mgg3.1843 PMC8922964 35150089

[89] Duis J, Skinner A, Carson R, Gouelle A, Annoussamy M, Silverman JL, Apkon S, Servais L, Carollo J. Quantitative measures of motor development in Angelman syndrome. Am J Med Genet A. 2023;191(7):1711–21. Epub 20230405. 10.1002/ajmg.a.63192. PubMed PMID: 37019838. 10.1002/ajmg.a.63192 PMC11068498 37019838

[90] Bird LM, Tan WH, Bacino CA, Peters SU, Skinner SA, Anselm I, Barbieri-Welge R, Bauer-Carlin A, Gentile JK, Glaze DG, Horowitz LT, Mohan KN, Nespeca MP, Sahoo T, Sarco D, Waisbren SE, Beaudet AL. A therapeutic trial of pro-methylation dietary supplements in Angelman syndrome. Am J Med Genet A. 2011;155A(12):2956–63. Epub 20111014. 10.1002/ajmg.a.34297. PubMed PMID: 22002941; PMCID: PMC3222728. 10.1002/ajmg.a.34297 PMC3222728 22002941

[91] Ciarlone SL, Grieco JC, D'Agostino DP, Weeber EJ. Ketone ester supplementation attenuates seizure activity, and improves behavior and hippocampal synaptic plasticity in an Angelman syndrome mouse model. Neurobiol Dis. 2016;96:38–46. Epub 20160818. 10.1016/j.nbd.2016.08.002. PubMed PMID: 27546058. 10.1016/j.nbd.2016.08.002 27546058

[92] Ciarlone SL, Wang X, Rogawski MA, Weeber EJ. Effects of the synthetic neurosteroid ganaxolone on seizure activity and behavioral deficits in an Angelman syndrome mouse model. Neuropharmacology. 2017;116:142–50. Epub 20161213. 10.1016/j.neuropharm.2016.12.009. PubMed PMID: 27986596. 10.1016/j.neuropharm.2016.12.009 27986596

[93] Adikari, Copping, J B, D C, KD F, JL S, JD A. Functional rescue in an Angelman syndrome model following treatment with lentivector transduced hematopoietic stem cells. Hum Gene Ther. 2021;In Press. 10.1093/hmg/ddab104 PMC8188406 33856035

[94] Leach PT, Crawley JN. Touchscreen learning deficits in Ube3a, Ts65Dn and Mecp2 mouse models of neurodevelopmental disorders with intellectual disabilities. Genes Brain Behav. 2018;17(6):e12452. Epub 2017/12/22. 10.1111/gbb.12452. PubMed PMID: 29266714; PMCID: PMC6013336. 10.1111/gbb.12452 PMC6013336 29266714

[95] Grieco JC, Bahr RH, Schoenberg MR, Conover L, Mackie LN, Weeber EJ. Quantitative Measurement of Communication Ability in Children with Angelman Syndrome. J Appl Res Intellect Disabil. 2018;31(1):e49-e58. Epub 20161219. 10.1111/jar.12305. PubMed PMID: 27990716. 10.1111/jar.12305 27990716

[96] Akula SK, McCullough KB, Weichselbaum C, Dougherty JD, Maloney SE. The trajectory of gait development in mice. Brain Behav. 2020;10(6):e01636. Epub 20200424. 10.1002/brb3.1636. PubMed PMID: 32333523; PMCID: PMC7303394. 10.1002/brb3.1636 PMC7303394 32333523

[97] Garrick JM, Costa LG, Cole TB, Marsillach J. Evaluating Gait and Locomotion in Rodents with the CatWalk. Curr Protoc. 2021;1(8): e220. 10.1002/cpz1.220.PubMedPMID:34370398;PMCID:PMC8363132. 34370398 10.1002/cpz1.220 PMC8363132

[98] Matas E, Maisterrena A, Thabault M, Balado E, Francheteau M, Balbous A, Galvan L, Jaber M. Major motor and gait deficits with sexual dimorphism in a Shank3 mutant mouse model. Mol Autism. 2021;12(1):2. Epub 20210119. 10.1186/s13229-020-00412-8. PubMed PMID: 33468258; PMCID: PMC7814442. 10.1186/s13229-020-00412-8 PMC7814442 33468258

[99] Rahn RM, Weichselbaum CT, Gutmann DH, Dougherty JD, Maloney SE. Shared developmental gait disruptions across two mouse models of neurodevelopmental disorders. J Neurodev Disord. 2021;13(1):10. Epub 20210320. 10.1186/s11689-021-09359-0. PubMed PMID: 33743598; PMCID: PMC7980331. 10.1186/s11689-021-09359-0 PMC7980331 33743598

[100] Gentile JK, Tan WH, Horowitz LT, Bacino CA, Skinner SA, Barbieri-Welge R, Bauer-Carlin A, Beaudet AL, Bichell TJ, Lee HS, Sahoo T, Waisbren SE, Bird LM, Peters SU. A neurodevelopmental survey of Angelman syndrome with genotype-phenotype correlations. J Dev Behav Pediatr. 2010;31(7):592–601. 10.1097/DBP.0b013e3181ee408e.PubMedPMID:20729760;PMCID:PMC2997715. 20729760 10.1097/DBP.0b013e3181ee408e PMC2997715

[101] Tan WH, Bacino CA, Skinner SA, Anselm I, Barbieri-Welge R, Bauer-Carlin A, Beaudet AL, Bichell TJ, Gentile JK, Glaze DG, Horowitz LT, Kothare SV, Lee HS, Nespeca MP, Peters SU, Sahoo T, Sarco D, Waisbren SE, Bird LM. Angelman syndrome: Mutations influence features in early childhood. Am J Med Genet A. 2011;155A(1):81–90. 10.1002/ajmg.a.33775.PubMedPMID:21204213;PMCID:PMC3563320. 21204213 10.1002/ajmg.a.33775 PMC3563320

[102] Mabb AM, Judson MC, Zylka MJ, Philpot BD. Angelman syndrome: insights into genomic imprinting and neurodevelopmental phenotypes. Trends Neurosci. 2011;34(6):293–303. Epub 20110517. 10.1016/j.tins.2011.04.001. PubMed PMID: 21592595; PMCID: PMC3116240. 10.1016/j.tins.2011.04.001 PMC3116240 21592595

[103] Lee HM, Clark EP, Kuijer MB, Cushman M, Pommier Y, Philpot BD. Characterization and structure-activity relationships of indenoisoquinoline-derived topoisomerase I inhibitors in unsilencing the dormant Ube3a gene associated with Angelman syndrome. Mol Autism. 2018;9:45. Epub 2018/08/25. 10.1186/s13229-018-0228-2. PubMed PMID: 30140420; PMCID: PMC6098585. 10.1186/s13229-018-0228-2 PMC6098585 30140420

[104] Kumar V, Joshi T, Vatsa N, Singh BK, Jana NR. Simvastatin Restores HDAC1/2 Activity and Improves Behavioral Deficits in Angelman Syndrome Model Mouse. Front Mol Neurosci. 2019;12:289. Epub 20191126. 10.3389/fnmol.2019.00289. PubMed PMID: 31849603; PMCID: PMC6901934. 10.3389/fnmol.2019.00289 PMC6901934 31849603

[105] Acosta MT, Kardel PG, Walsh KS, Rosenbaum KN, Gioia GA, Packer RJ. Lovastatin as treatment for neurocognitive deficits in neurofibromatosis type 1: phase I study. Pediatr Neurol. 2011;45(4):241–5. 10.1016/j.pediatrneurol.2011.06.016. PubMed PMID: 21907886. 21907886 10.1016/j.pediatrneurol.2011.06.016

[106] Bearden CE, Hellemann GS, Rosser T, Montojo C, Jonas R, Enrique N, Pacheco L, Hussain SA, Wu JY, Ho JS, McGough JJ, Sugar CA, Silva AJ. A randomized placebo-controlled lovastatin trial for neurobehavioral function in neurofibromatosis I. Ann Clin Transl Neurol. 2016;3(4):266–79. Epub 20160222. 10.1002/acn3.288. PubMed PMID: 27081657; PMCID: PMC4818747. 10.1002/acn3.288 PMC4818747 27081657

[107] Payne JM, Barton B, Ullrich NJ, Cantor A, Hearps SJ, Cutter G, Rosser T, Walsh KS, Gioia GA, Wolters PL, Tonsgard J, Schorry E, Viskochil D, Klesse L, Fisher M, Gutmann DH, Silva AJ, Hunter SJ, Rey-Casserly C, Cantor NL, Byars AW, Stavinoha PL, Ackerson JD, Armstrong CL, Isenberg J, O'Neil SH, Packer RJ, Korf B, Acosta MT, North KN, Consortium NFCT. Randomized placebo-controlled study of lovastatin in children with neurofibromatosis type 1. Neurology. 2016;87(24):2575–84. Epub 20161109. 10.1212/WNL.0000000000003435. PubMed PMID: 27956565; PMCID: PMC5207004. 10.1212/WNL.0000000000003435 PMC5207004 27956565

[108] Thurman AJ, Potter LA, Kim K, Tassone F, Banasik A, Potter SN, Bullard L, Nguyen V, McDuffie A, Hagerman R, Abbeduto L. Controlled trial of lovastatin combined with an open-label treatment of a parent-implemented language intervention in youth with fragile X syndrome. J Neurodev Disord. 2020;12(1):12. Epub 20200422. 10.1186/s11689-020-09315-4. PubMed PMID: 32316911; PMCID: PMC7175541. 10.1186/s11689-020-09315-4 PMC7175541 32316911

[109] Caku A, Pellerin D, Bouvier P, Riou E, Corbin F. Effect of lovastatin on behavior in children and adults with fragile X syndrome: an open-label study. Am J Med Genet A. 2014;164A(11):2834–42. Epub 20140924. 10.1002/ajmg.a.36750. PubMed PMID: 25258112. 10.1002/ajmg.a.36750 25258112

