# Supplementary material for: Acute administration of lovastatin had no pronounced effect on motor abilities, motor coordination, gait nor simple cognition in a mouse model of Angelman syndrome
Source: J Neurodev Disord. 2025 May 17;17:27. doi: 10.1186/s11689-025-09616-6 (PMC12085040; doi:10.1186/s11689-025-09616-6)

**
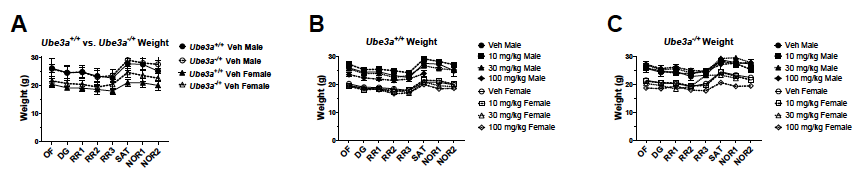
**

**Figure S1. Mouse weights throughout testing battery.**

(A) Vehicle treated *Ube3a*^-/+^ mouse weights were not significantly different from vehicle treated *Ube3a*^+/+^ mice. (B) Treating with lovastatin had no significant effect on weight when compared to vehicle treated *Ube3a*^+/+^ mice. (C) Weights of *Ube3a*^-/+^ mice were not affected by any dose of lovastatin. Weights were taken prior to each behavioral task. Data analyzed with Repeated Measures ANOVA by treatment within genotype. Abbreviations: OF - open field task; DG – DigiGait; RR1 – rotarod day 1; RR2 – rotarod day 2; RR3 – rotarod day 3; SAT – spontaneous alternation task; NOR1 – novel object recognition day 1; NOR2 – novel object recognition day 2.


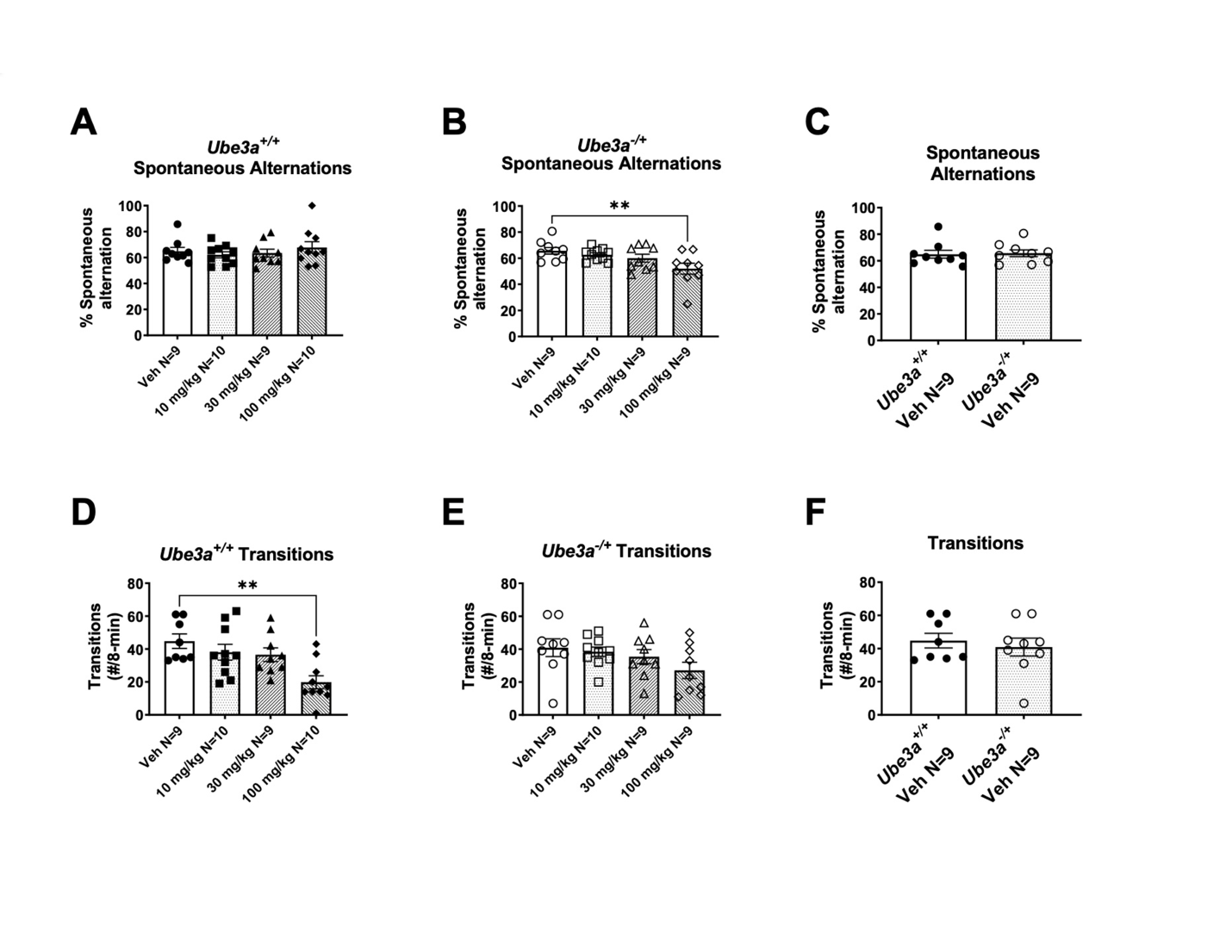


**Figure S2. Lovastatin reduced triads in *Ube3a^-/+^* and transitions in *Ube3a^+/+^* mice, during the spontaneous alternation Y- maze assay.**

(A) Treating *Ube3a^+/+^* mice with any dose of lovastatin had no effect on the percentage of triad alternations. (B) A dose of 100 mg/kg significantly reduced the percentage of triad alternations made by *Ube3a^-/+^* mice. (C) Cognitive deficits in vehicle treated *Ube3a^-/+^* mice were not observed when compared to *Ube3a^+/+^* mice by triad alterations. (D) Treatment of *Ube3a^+/+^* mice with 100 mg/kg of lovastatin significantly reduced the number of total transitions made in the Y-maze. (E) No difference in number of total transitions was observed when *Ube3a^-/+^* mice were treated with any dose of lovastatin. (F) Vehicle treated *Ube3a^-/+^* and *Ube3a^+/+^* mice displayed no differences in total number of transitions over the 8-minute task which does not demonstrate a working memory deficit of *Ube3a^-/+^* mice when compared to *Ube3a^+/+^* mice. (A-C, D-F). Data are expressed as mean +/− S.E.M. * < 0.05, Two-Way ANOVA by genotype and treatment. (D, H) * p < 0.05, T-test of novel versus familiar.

**Table S1. Two-Way Repeated Measures ANOVA for Open Field Total Distance.**

WT Mice Total Activity Over Time and 3 Doses of Lovastatin: Supplementary Table 1

| Dunnett's multiple comparisons test | 95.00% CI of diff. | Significant? | Summary | Adjusted P Value |
| --- | --- | --- | --- | --- |
|  |  |  |  |  |
| 1-5 |  |  |  |  |
| WT VEH vs. WT 10 10mg/kg | -139.8 to 649.6 | No | ns | 0.294 |
| WT VEH vs. WT 30 mg/kg | 110.5 to 899.9 | Yes | ** | 0.0078 |
| WT VEH vs. WT 100 mg/kg | 544.6 to 1334 | Yes | **** | <0.0001 |
|  |  |  |  |  |
| 6-10 |  |  |  |  |
| WT VEH vs. WT 10 10mg/kg | -125.8 to 663.6 | No | ns | 0.2537 |
| WT VEH vs. WT 30 mg/kg | 80.23 to 869.6 | Yes | * | 0.0135 |
| WT VEH vs. WT 100 mg/kg | 165.5 to 954.9 | Yes | ** | 0.0027 |
|  |  |  |  |  |
| 11-15 |  |  |  |  |
| WT VEH vs. WT 10 10mg/kg | -171.6 to 617.8 | No | ns | 0.3999 |
| WT VEH vs. WT 30 mg/kg | -25.97 to 763.4 | No | ns | 0.0731 |
| WT VEH vs. WT 100 mg/kg | 99.83 to 889.2 | Yes | ** | 0.0095 |
|  |  |  |  |  |
| 16-20 |  |  |  |  |
| WT VEH vs. WT 10 10mg/kg | -129.1 to 660.3 | No | ns | 0.2629 |
| WT VEH vs. WT 30 mg/kg | -103.4 to 686.0 | No | ns | 0.1977 |
| WT VEH vs. WT 100 mg/kg | -10.77 to 778.6 | No | ns | 0.0587 |
|  |  |  |  |  |
| 21-25 |  |  |  |  |
| WT VEH vs. WT 10 10mg/kg | -216.2 to 573.2 | No | ns | 0.5762 |
| WT VEH vs. WT 30 mg/kg | -128.5 to 660.9 | No | ns | 0.2612 |
| WT VEH vs. WT 100 mg/kg | 19.73 to 809.1 | Yes | * | 0.0369 |
|  |  |  |  |  |
| 26-30 |  |  |  |  |
| WT VEH vs. WT 10 10mg/kg | -287.2 to 502.2 | No | ns | 0.8565 |
| WT VEH vs. WT 30 mg/kg | -247.4 to 542.0 | No | ns | 0.7067 |
| WT VEH vs. WT 100 mg/kg | -112.3 to 677.1 | No | ns | 0.2188 |

**AS Mice Total Activity Over Time Over Time and 3 Doses of Lovastatin: Supplementary Table 2**

**Table S2. Two-Way Repeated Measures ANOVA for Open Field Horizontal Activity.**

**
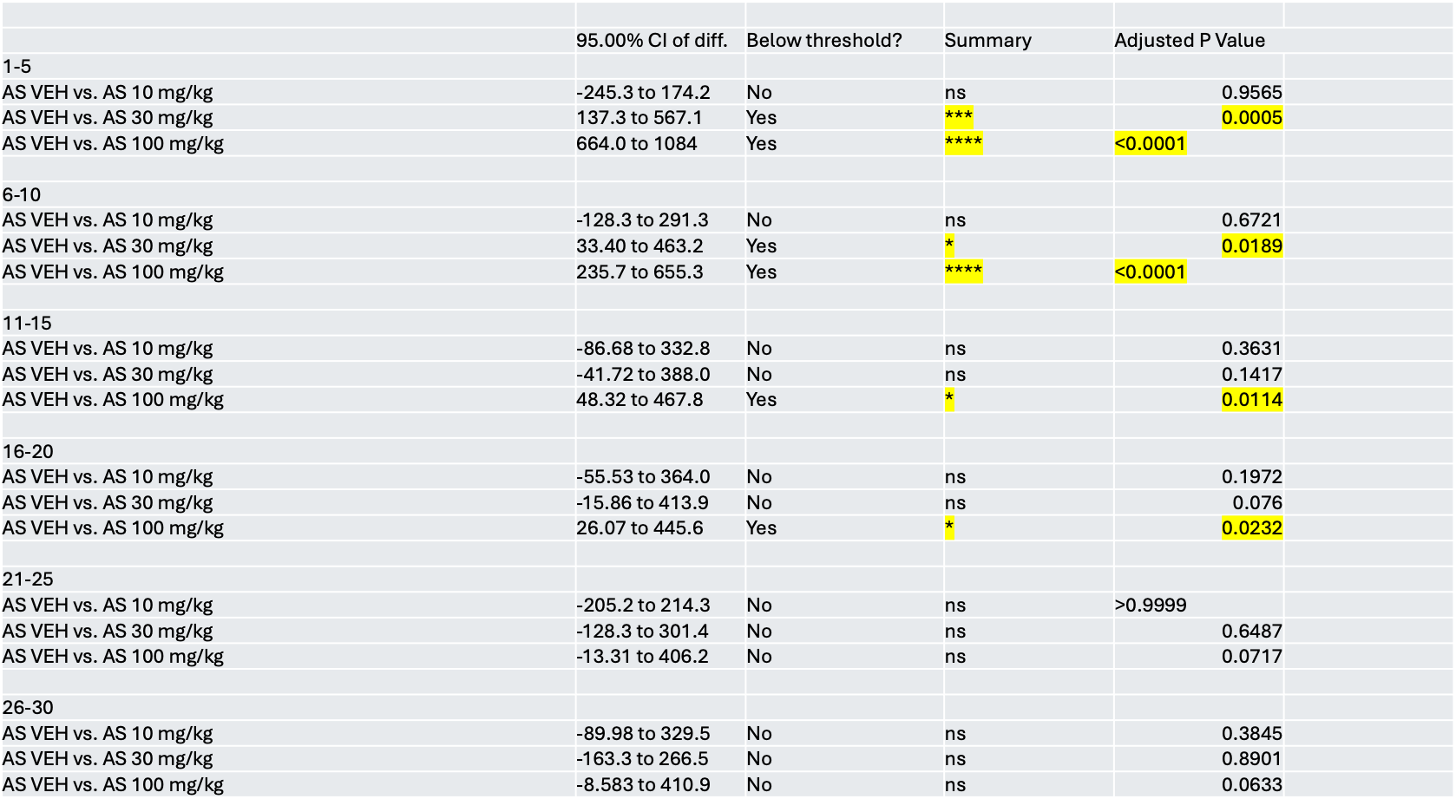
**

**Table S3. WT Mice Horizontal Activity Across 3 Doses of Lovastatin : Supplementary Table 3.**


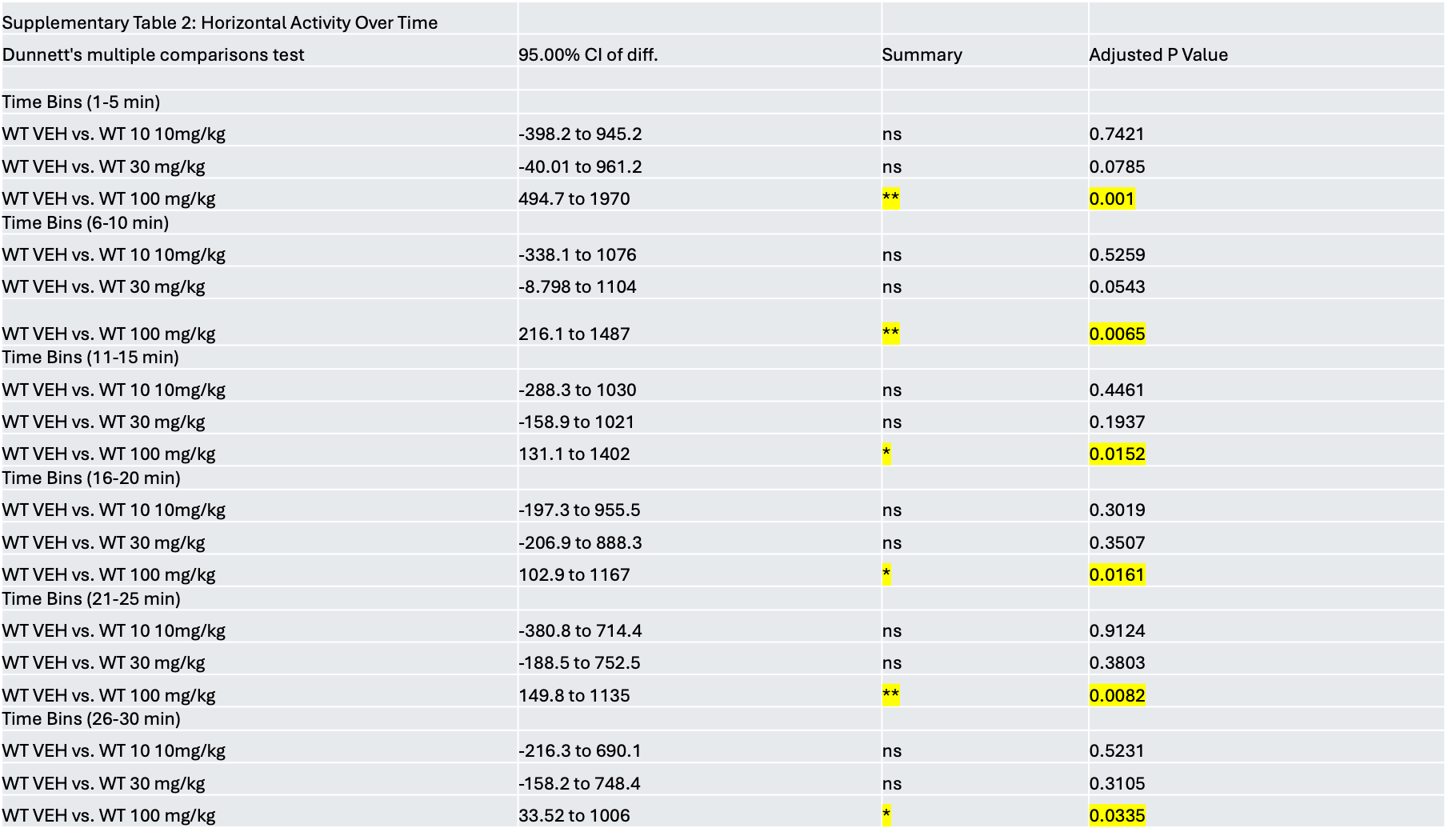


**Table S4. AS Mice Horizontal Activity Across 3 Doses of Lovastatin: Supplementary Table 4.**


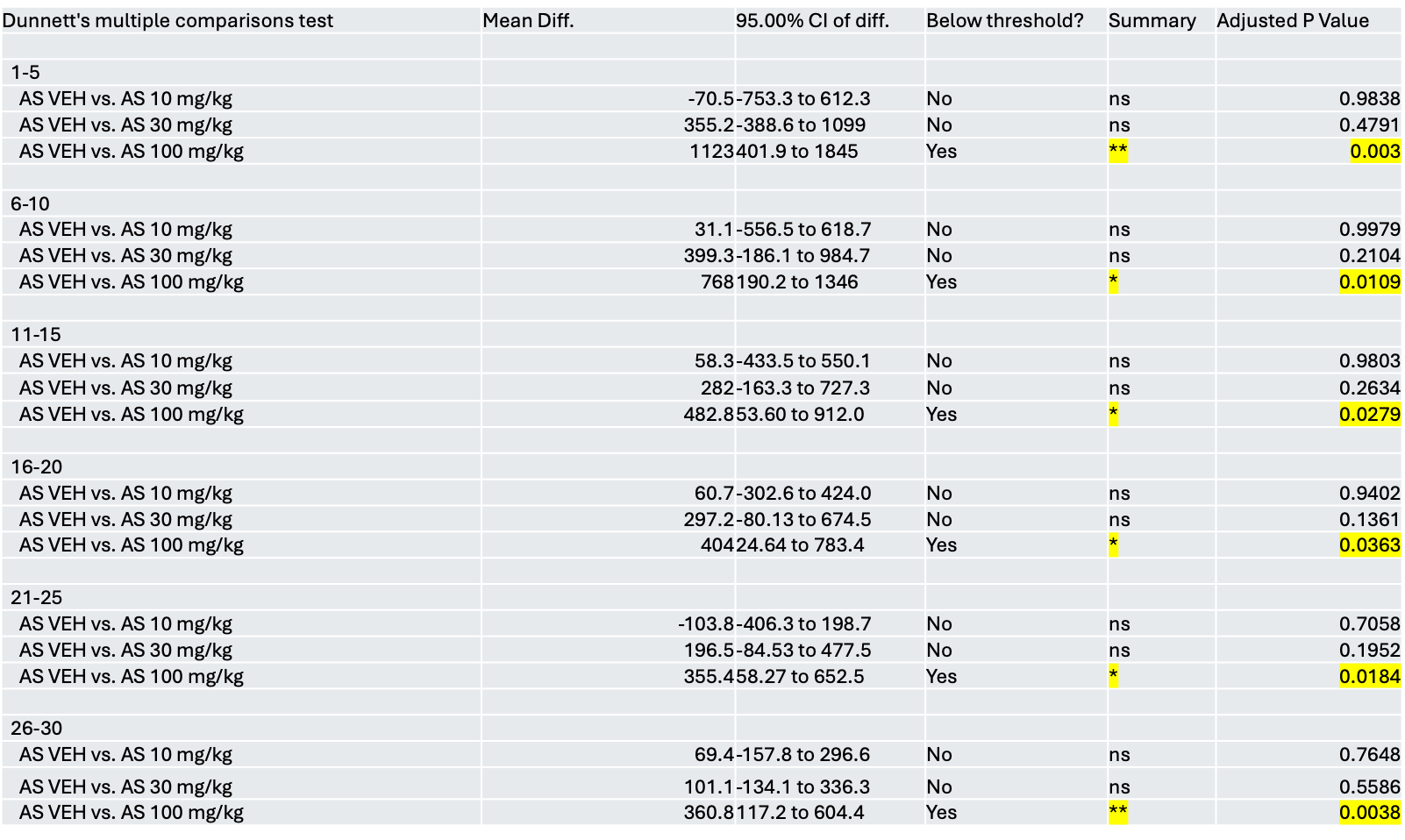


**Table S5. WT Mice Summed Total Activity: Supplementary Table S5**


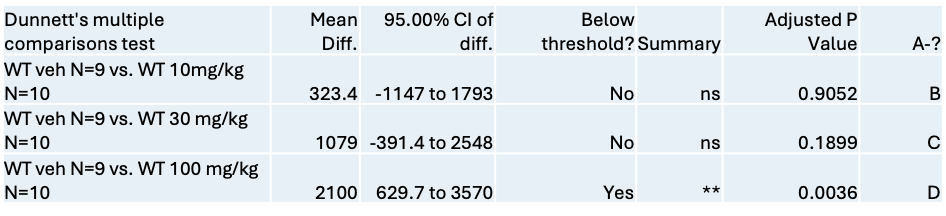


**Table S6. AS Mice Summed Total Activity: Supplementary Table S6**


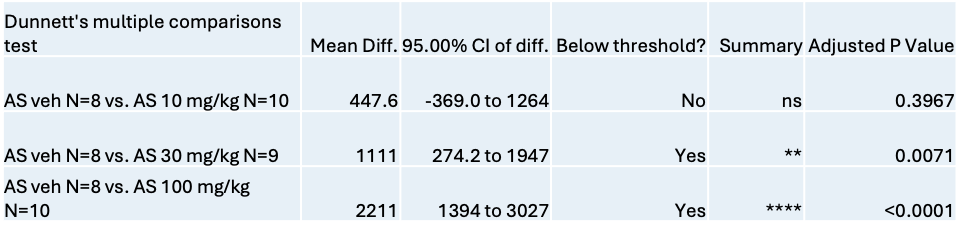


**Table S7. WT Mice Summed Vertical Activity: Supplementary Table S7**


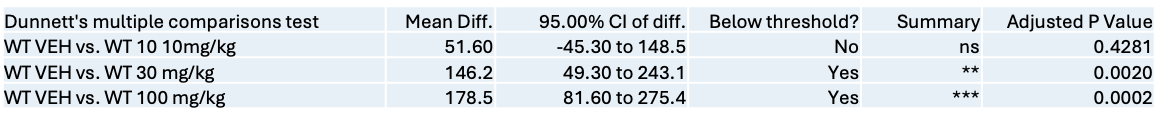


**Table S8. AS Mice Summed Vertical Activity: Supplementary Table S8**


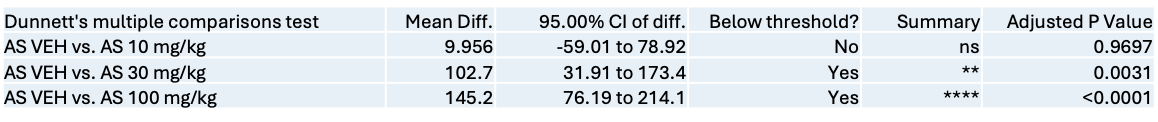

Supplement: Supplementary file 1 — Supplementary Material 1. [file 11689_2025_9616_MOESM1_ESM.docx]
